# A preliminary comparison of prosthetic socket liner strain determined using digital image correlation and finite element analysis

**DOI:** 10.1371/journal.pone.0353881

**Published:** 2026-07-14

**Authors:** Mohammadreza Freidouny, Michael L. Madigan, Carson Squibb, Masaki Hada, Abbie Bailey, Trevor Johnson, Brian Kaluf, Michael K. Philen

**Affiliations:** 1 Grado Department of Industrial & Systems Engineering, Virginia Tech, Blacksburg, Virginia, United States of America; 2 Kevin T. Crofton Department of Aerospace and Ocean Engineering, Virginia Tech, Blacksburg, Virginia, United States of America; 3 Virginia Prosthetics and Orthotics, Christiansburg, Virginia, United States of America; 4 Salt Lake City Research Hub, Ottobock, Salt Lake City, Utah, United States of America; Politecnico di Torino, ITALY

## Abstract

Improving prosthetic socket fit remains an important issue among prosthesis users. Understanding strains at limb-socket interface may help prosthetists improve socket fit. Previous studies have used finite element analysis (FEA) to predict stresses and strains at limb-socket interface. However, the selection of FEA model material properties as well as the validation of FEA strain results remain challenging. Digital image correlation (DIC) is a well-accepted optical technique that is widely used in experimental mechanics to noninvasively measure strains and can be used to validate FEA predictions. The goal of this feasibility study was to demonstrate the use of DIC to measure prosthesis liner strains of a unilateral transtibial prosthesis user and conduct a preliminary comparison of these strains with those estimated from an FEA model. One participant with a transtibial residual limb was recruited. Liner strains of the residual limb during two stepping tasks were measured with DIC. An FEA model of the residual limb was also developed, and its estimates of liner strains were compared with DIC measurements. Results showed that mean and probability distributions of principal strain values measured by DIC were in moderate to good agreement with FEA estimations. For example, DIC measured mean maximum and minimum principal strains of 0.041 and 0.021 in the anterior aspect of the residual limb when it was being vertically loaded, and FEA estimated mean maximum and minimum principal strains of 0.051 and 0.027 in the same region. The Bhattacharyya coefficient (a measure of overlap between two probability distributions, with values approaching 1 indicating high similarity) exceeded 0.90 for principal strain distributions between DIC and FEA on the anterior aspect of the residual limb during both tasks. These findings demonstrate that DIC can be used to refine FEA model material properties and results. As such, this methodology may help to advance the design of prosthetic sockets; however, as this study involved a single participant, findings are preliminary and intended to demonstrate methodological feasibility rather than generalize across the broader population of prosthesis users.

## Introduction

Prosthesis users indicate the most important issue with their prosthesis is socket fit [1]. Poor socket fit can result from high stresses and strains at the limb-socket interface [2,3]. The challenge of measuring these stresses and strains has limited our understanding of factors associated with socket fit [4]. With this in mind, in-vivo methods that involve discrete transducers and sensors have been used to measure stresses at the limb-socket interface [5–9]. However, these methods have several technical challenges such as being expensive, bulky, non-linear, temperature sensitive, and having limited spatial resolution [4,8].

Finite element analysis (FEA) is a powerful tool for predicting stresses and strains [10]. Dickinson et al. [11] identified up to 45 studies from 2000 to 2016 that developed FEA models of lower residual limbs to estimate stresses and strains at the limb-socket interface or within the soft tissues. However, the challenge of experimentally measuring these stresses and strains has made the important step of validating FEA results difficult [12]. As a result, FEA model predictions of stresses and strains on the residual limb of lower limb amputees vary across different studies. For example, Lee and Ming [13] estimated peak normal stress of 230 kPa at the patellar tendon region of a transtibial residual limb during single-leg standing while Plesec and Harih [14] estimated peak normal stress of 106 kPa at the same region during the same task when using a higher body weight. Furthermore, FEA predictions of interface stresses have shown average errors of up to 12 kPa from experimental measurements [15,16], while skin shear stresses ranging from 4 to 23 kPa under cyclic loadings have been associated with skin damage risk [17], underscoring the importance of accurate FEA predictions for safe socket design. The use of experimental methods to directly measure stresses and strains with high spatial resolution and accuracy would enable the validation of FEA predictions.

Digital image correlation (DIC) is a well-accepted optical technique that is widely used in experimental mechanics to measure full-field surface displacements and strains [18,19]. DIC involves applying a speckled pattern on the surface of interest, capturing changes in this pattern over time using high-resolution digital cameras as loads are applied, and processing these changes in pattern using computational algorithms to determine strains. DIC has the potential to validate and improve FEA models of prosthetic sockets and residual limbs, yet only a limited number of studies have attempted to use it in prosthesis-related applications. For example, Moerman et al. [20] used DIC to estimate the mechanical properties of a silicone-gel material during indentation tests, representing human soft tissue, and then used these data to improve the corresponding FEA predictions. Saey et al. [21] used DIC to measure strain on the outer surface of a transtibial prosthetic socket under cyclic load simulating gait, with the goal of assessing socket mechanical properties in the design process. Solav et al. [22] used DIC to measure skin strain on a bare transtibial residual limb, without donning a liner or socket, during controlled seated tasks including knee flexion/extension and other muscle contractions. We are not aware of any studies that attempted to measure skin strain while an individual used their prosthesis during a load-bearing activity such as standing or walking.

The goals of this single-participant, feasibility study were to first demonstrate the use of DIC to measure the prosthesis liner strain of a unilateral transtibial prosthesis user during two stepping tasks, and then to conduct a preliminary comparison of these strains with those predicted from an FEA model. The results of this study will help establish a DIC methodology for studying lower limb prostheses that can inform the selection of FEA model material properties as well as validating FEA results. Thus, DIC has the potential to improve FEA models as well as the understanding of factors associated with prosthetic socket fit and comfort.

## Methods

### Human subject testing

One male participant (46 years old, 86 kg body mass) with a unilateral transtibial amputation as a result of trauma seven years prior was recruited. The participant was selected based on: a) having a below knee amputation at least one year prior; b) having a well-fitting prosthesis that was used daily; and c) schedule availability. The participant was recruited on December 1, 2023, and data collection was completed on the same day. The participant provided written informed consent prior to participation, and the study was approved by Virginia Tech Institutional Review Board (IRB 21–764).

**Fig 1:**
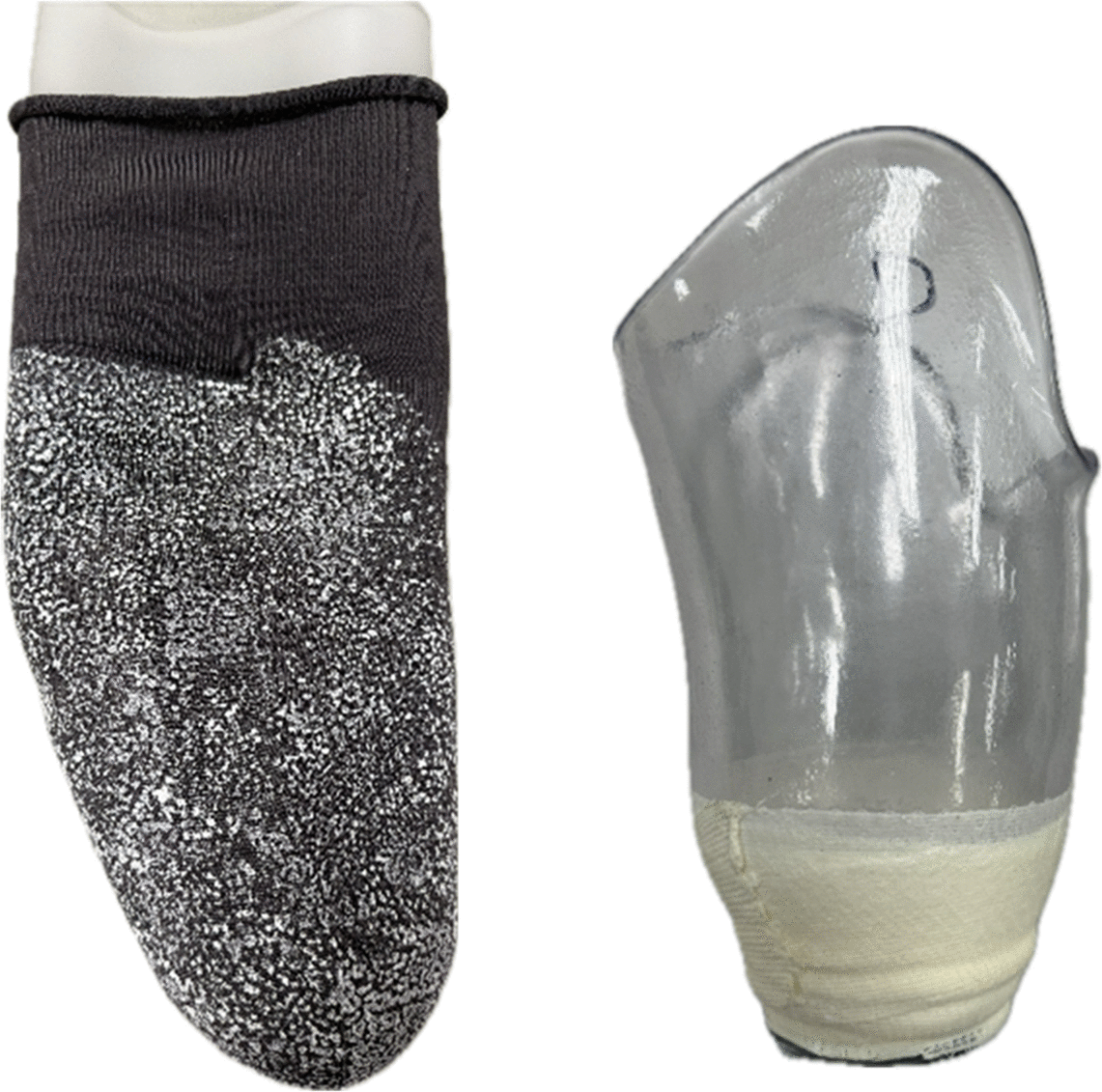
Lateral views of the speckled liner (left) and diagnostic socket (right).

Upon arrival, the participant removed his personal prosthesis and sat in a chair for 20 minutes to allow his residual limb volume to equilibrate. During this time, a custom-fabricated total surface bearing clear diagnostic socket was attached to his personal daily-use pylon and prosthetic foot by a certified prosthetist. Approximate lengths of the tibia and fibula were also palpated and obtained using a tape measure for later use with the FEA model. After 20 minutes, the bare residual limb was scanned using a Peel 3D scanner (Creaform, Lévis, Canada). The participant then donned a liner (6Y512 Uneo, Otto Bock, Duderstadt, Germany) and the diagnostic socket (Fig 1) and walked for approximately three minutes to acclimate to the socket. Prior to the experimental session, the exterior of the liner had been speckled using a 3D-printed stamp with 1.5 mm diameter speckles that were pseudo-randomly distributed spatially. The speckles were created using a fabric dye mixed with paint thickener.

**Fig 2:**
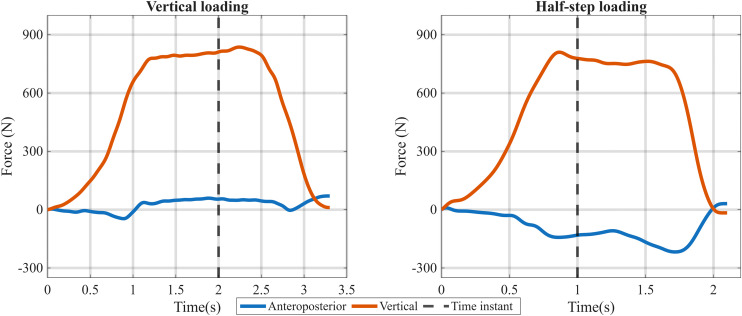
Anteroposterior and vertical components of ground reaction forces during vertical loading task (left) and the half-step task (right). The vertical dashed line indicates the time point at which the strain data was compared between DIC and FEA. A negative value in the anteroposterior force indicates the ground reaction force was directed posteriorly.

**Fig 3:**
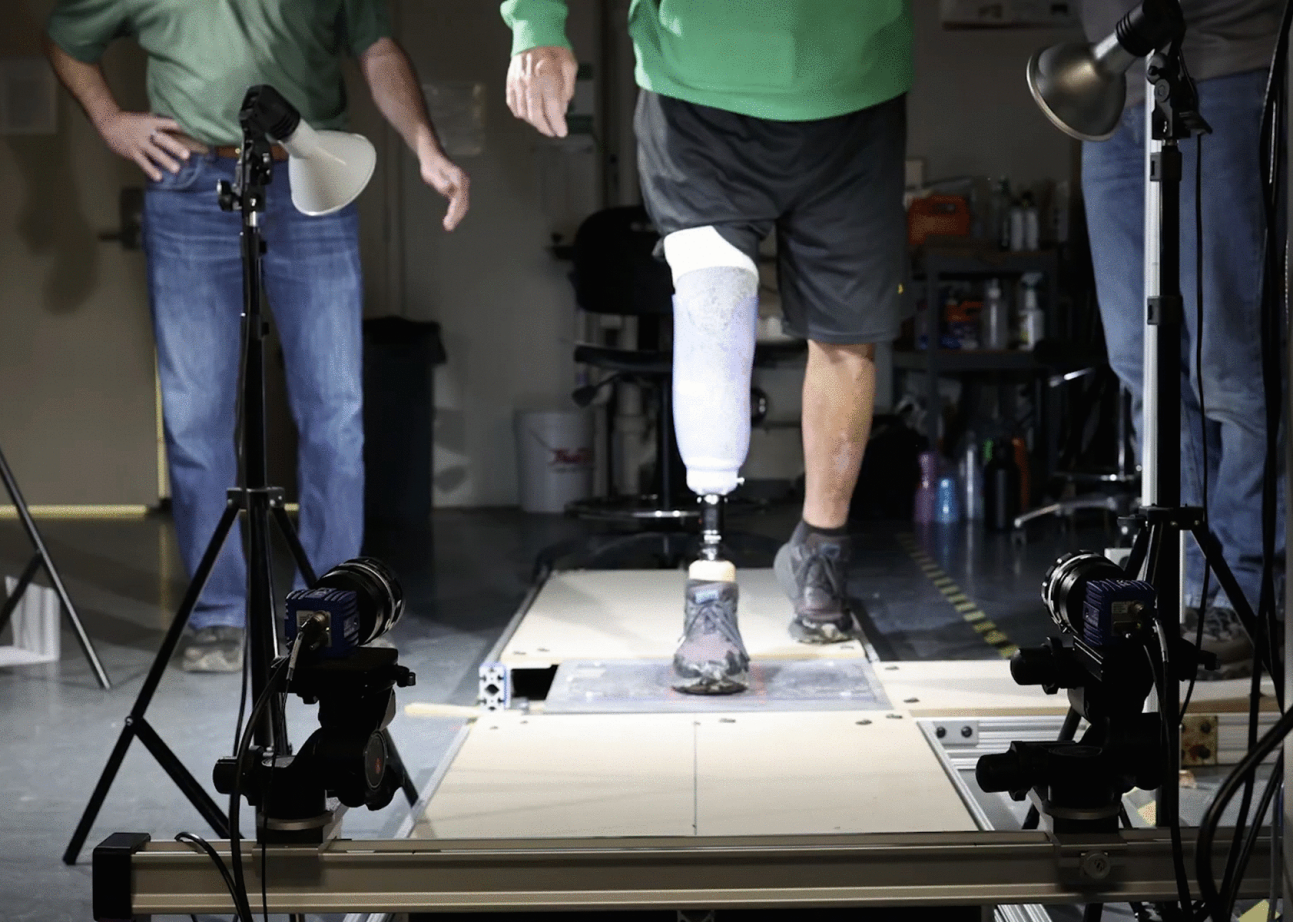
Experimental setup for collecting liner strain and ground reaction forces during the half-step task.

The participant then completed two stepping tasks for analysis including a vertical loading task and a half-step loading task. During the vertical loading task, the participant initially stood on his unaffected leg with his prosthetic foot hovering just above a forceplate (FP4550−08, Bertec Corp., Columbus, OH, USA). When indicated by the researcher, the participant slowly lowered his prosthetic foot onto the forceplate, gradually applied more of his body weight onto his prosthetic foot until his full body weight was supported, then reversed the loading until the prosthetic foot was off the forceplate. The goal of this task was to apply primarily vertical loading to the prosthetic foot (Fig 2) with minimal transverse loading or changes in foot position and kinematics. During the half-step task, the participant stood in a tandem stance with the unaffected foot in front of the prosthetic foot and within a single-step distance from the forceplate. When indicated by the researcher, the participant initiated a step onto the forceplate by his prosthetic foot, similar to heel contact during gait, and gradually shifted full-body weight onto the prosthetic foot (Fig 3), then slowly pushed off to move backward and return to the starting position. The goal of this task was to apply both vertical and anteroposterior loading to the foot (Fig 2), but in a more controlled manner than normal gait. Because only two DIC cameras were available (see details below), two trials of each task were completed with both cameras used to capture video on the anterior aspect of the limb during the first trials and both cameras used to capture video on the lateral aspect of the limb during the second trials. This approach could introduce potential inconsistencies between trials due to possible changes in loading conditions or prosthetic foot positioning. To minimize inconsistencies between trials, participants were provided with verbal instructions and visual markers on the forceplate. Moreover, future studies should investigate intra-trial variability of strain measurements.

### Digital image correlation

**Fig 4:**
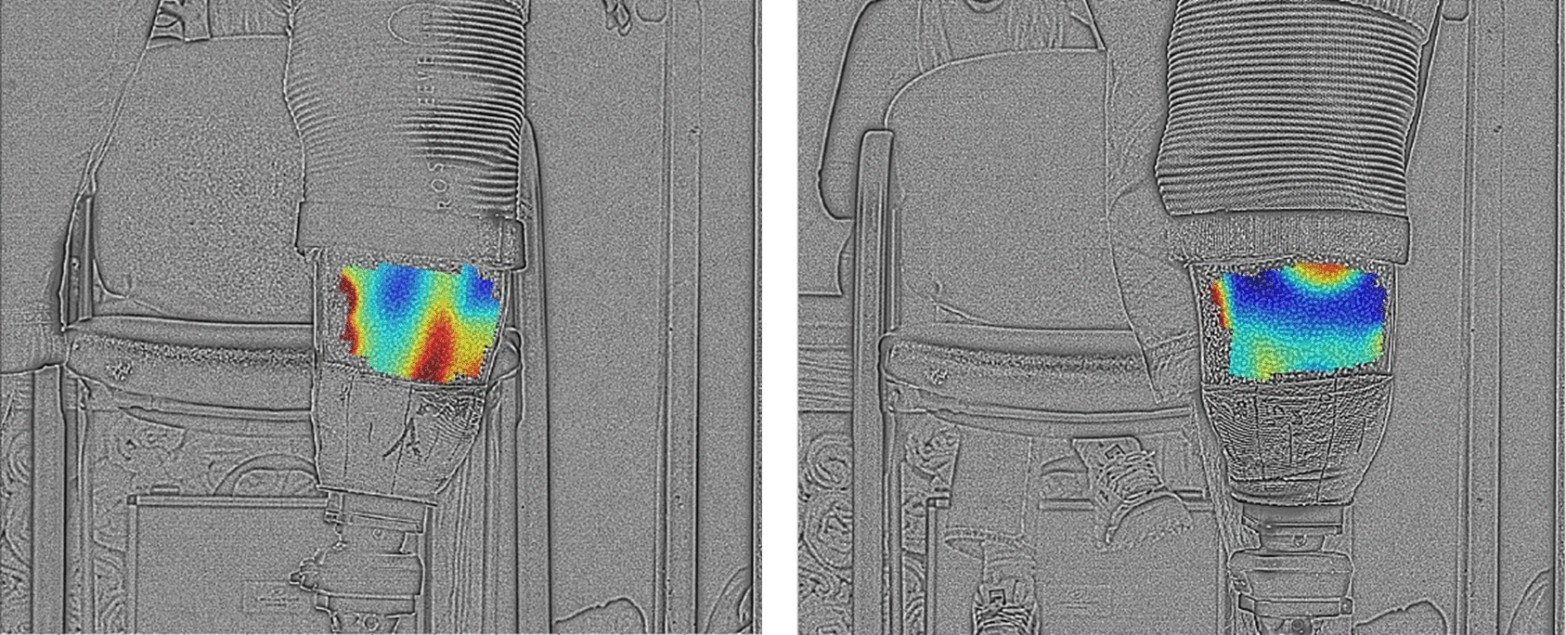
An illustration of the anterior (left) and lateral (right) views of the measurable strain region on the residual limb within the DaVis software.

Two DIC cameras (Imager M-lite 5M, LaVision, Göttingen, Germany), each equipped with a lens (Color Skopar 28 mm f/2.8, Voigtländer, Braunschweig, Germany) to provide appropriate focal distance and field of view, were mounted at an approximate height of 20 cm above the forceplate and at a distance of 160 cm from the forceplate. The cameras had a focal length of 29 mm and a pixel size of 0.00345 mm. Camera calibration was performed using a pinhole fit model with a fit error of 0.0538 pixels. One pair of light projectors was used as the only light in the room and were adjusted in their position and orientation to minimize light reflection on the socket surface. Liner surface strains were calculated from the DIC recordings using DaVis 10 software (LaVision, Göttingen, Germany) with a subset size of 49 pixels and a step size of 16 pixels. DIC image processing included intensity normalization to have a uniform image contrast and a moving average filter with a length of 5 pixels to reduce noise. The height of the measurable strain region was approximately 7 cm and just distal to the fibular head (Fig 4). This height was limited due to the distal fiberglass wrap applied to maintain structural integrity and the proximal sealing sleeve used to maintain suspension of the prosthesis. The width of the measurable strain region in the circumferential direction was approximately 8 cm and limited due to our use of only two DIC cameras.

### Finite element analysis

**Fig 5:**
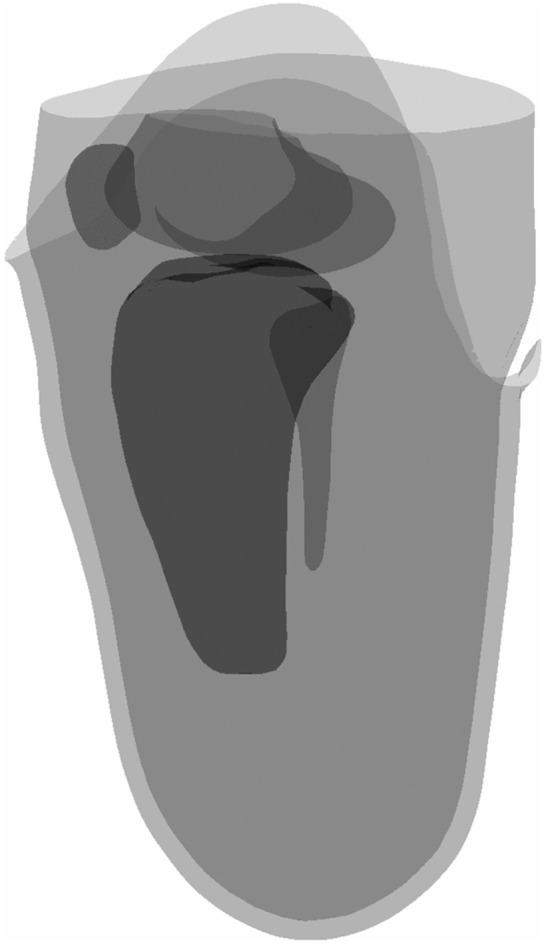
Lateral view of the FEA model of the residual limb within the socket.

The point cloud data of the residual limb from Peel 3D scanner was imported into MeshLab [23], and the three-dimensional geometry of the limb was generated. The three-dimensional geometry of the prosthetic socket was provided to the investigators by the socket fabricator as a computer file. The liner was modeled as a 5.5 mm-thickness shell added to the outer surface of the residual limb. The use of computed tomography or magnetic resonance imaging to obtain tibia and fibula bone geometry was not feasible for this study. Alternatively, we incorporated bone structures from an open-access model [24] and modified their size and position to align with our initial tape measurements. All 3D geometries were then assembled, and a nonlinear quasi-static FEA model of the residual limb with liner and prosthetic socket (Fig 5) was generated with ABAQUS (Dassault Systèmes, RI, USA) to determine the maximum and minimum principal strains on the liner surface at two time points of interest (Fig 3), for comparison with the strains determined from DIC.

Soft tissues, including the skin, muscles, ligaments, fat, and tendons, were all modeled as linear elastic (Table 1). Three values of Young’s modulus (0.1, 0.2, and 0.3 MPa) with a Poisson’s ratio of 0.49 used by previous studies [11,25] were evaluated for all soft tissues to identify the best match with DIC measurements. A Young’s modulus of 0.3 MPa provided the closest liner strain match with DIC measurements and was selected for the final model. The bones, liner, and socket were also modeled as linear elastic materials as in prior studies (Table 1). A frictionless surface-to-surface contact was applied at the liner-socket interface. Liner-skin and bone-soft tissue interactions were defined to be tied. Using the available element libraries in ABAQUS, a free meshing technique using four-node tetrahedral elements (C3D4) was implemented for the soft tissue. The liner was meshed with four-node shell elements (S4). The socket and bones were meshed with 3-node shell (S3) and four-node tetrahedral (C3D4) elements, respectively.

**Table 1:** Material properties and elements used for modelling FEA components.

FEA Model Component	Material Properties	Elements
	*E* (MPa), *ν*	
Soft tissue	0.3, 0.49	C3D4
Liner	0.17, 0.49 [26]	S4
Bones	3000, 0.30 [27]	C3D4
Socket	2100, 0.33 [28]	S3

**Fig 6:**
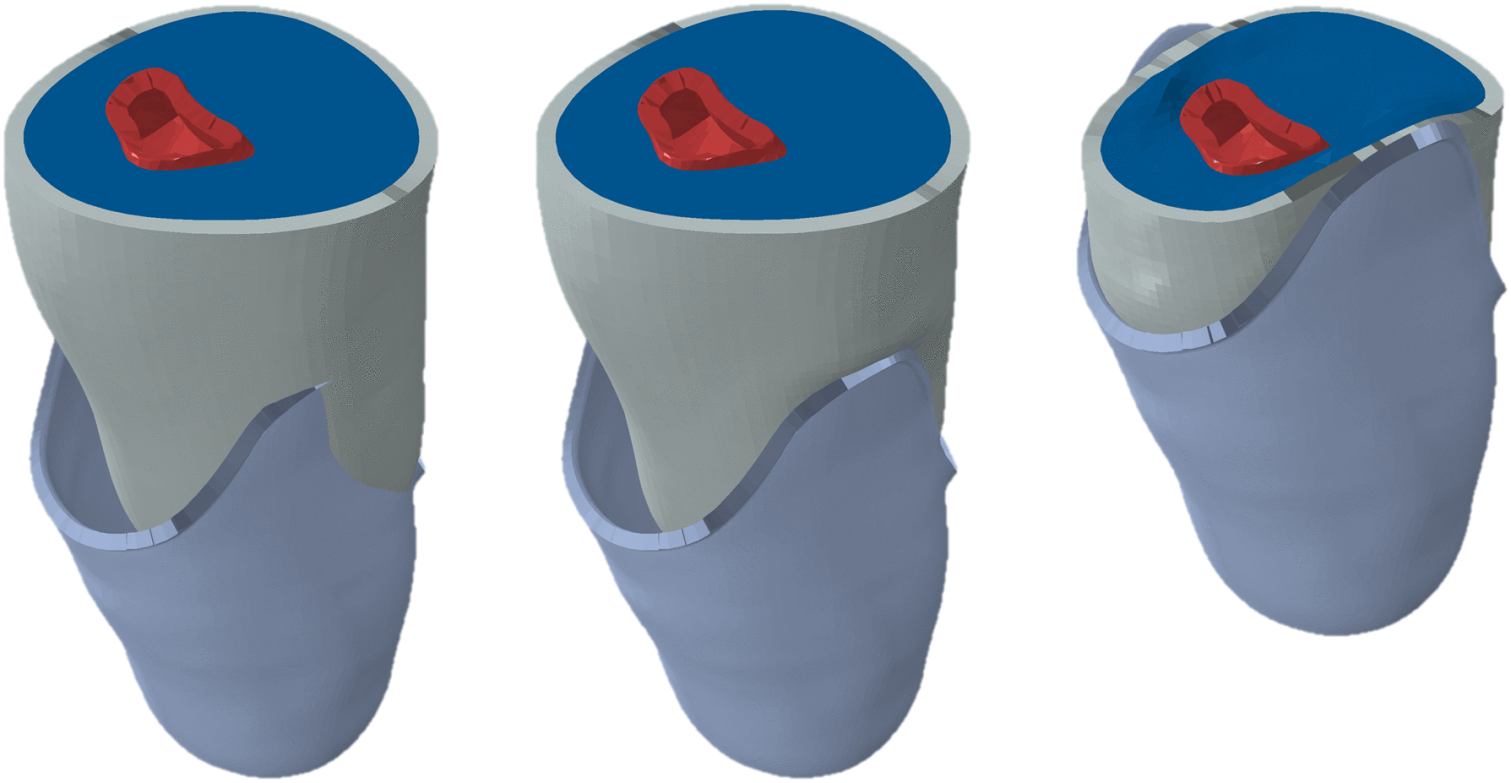
Simulation phases of the donning process in the FEA model. The sequence shows (from left to right): initial assembly of the residual limb and socket before FEA solving, adjusted alignment to eliminate overlap, and final placement of the residual limb within the socket.

After manually positioning the socket and residual limb to be longitudinally aligned and have minimal overlap, ABAQUS solver adjusted the shape of the residual limb and liner to eliminate any initial overlap with the socket surface. Once the overlap was removed, a vertical displacement boundary condition was applied to the socket to fully don the residual limb within the socket (Fig 6). After the donning process, vertical and anteroposterior forces and the resultant sagittal plane moment over time, calculated from ground reaction forces, were applied to a reference node at the tibia head. To simplify the loading conditions, moments in the frontal and transverse planes were neglected [29,30] and the location of the center of pressure of the ground reaction forces was assumed to be aligned with the tibial axis. Knee angle was determined using sagittal plane captured videos during both tasks.

### DIC and FEA comparison

Due to differences in spatial resolution and mesh discretization, point-wise comparison of DIC and FEA strain fields was not feasible. Gomez et al. [31] faced a similar challenge when comparing FEA strain fields with medical image measurements; they addressed this by comparing strain histograms rather than point-wise values. To compare our DIC measurements with FEA model results, we compared strain mean, standard deviation (SD), and strain distributions within the measurable strain region. To compare strain distributions, probability distribution histograms of DIC and FEA strains were generated and compared using the Bhattacharyya coefficient (BC), a measure of the overlap between two probability distributions, where a value of 1 indicates complete overlap and 0 indicates no overlap [32].

## Results

**Fig 7:**
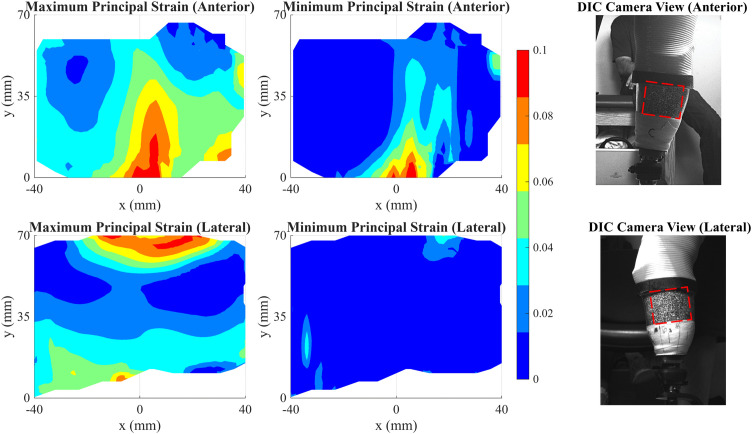
DIC measurements of principal strains on the liner surface during the vertical loading task when the vertical force was 810 N and the anteroposterior force was 50 N.

**Fig 8:**
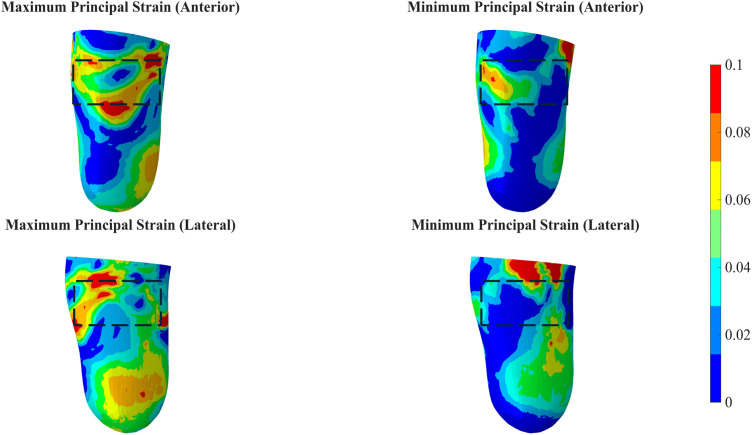
FEA measurements of principal strains on the liner surface when the vertical force was 810 N and the anteroposterior force was 50 N during the vertical loading task. Black box indicates the approximate location of DIC measurable strain region.

**Fig 9:**
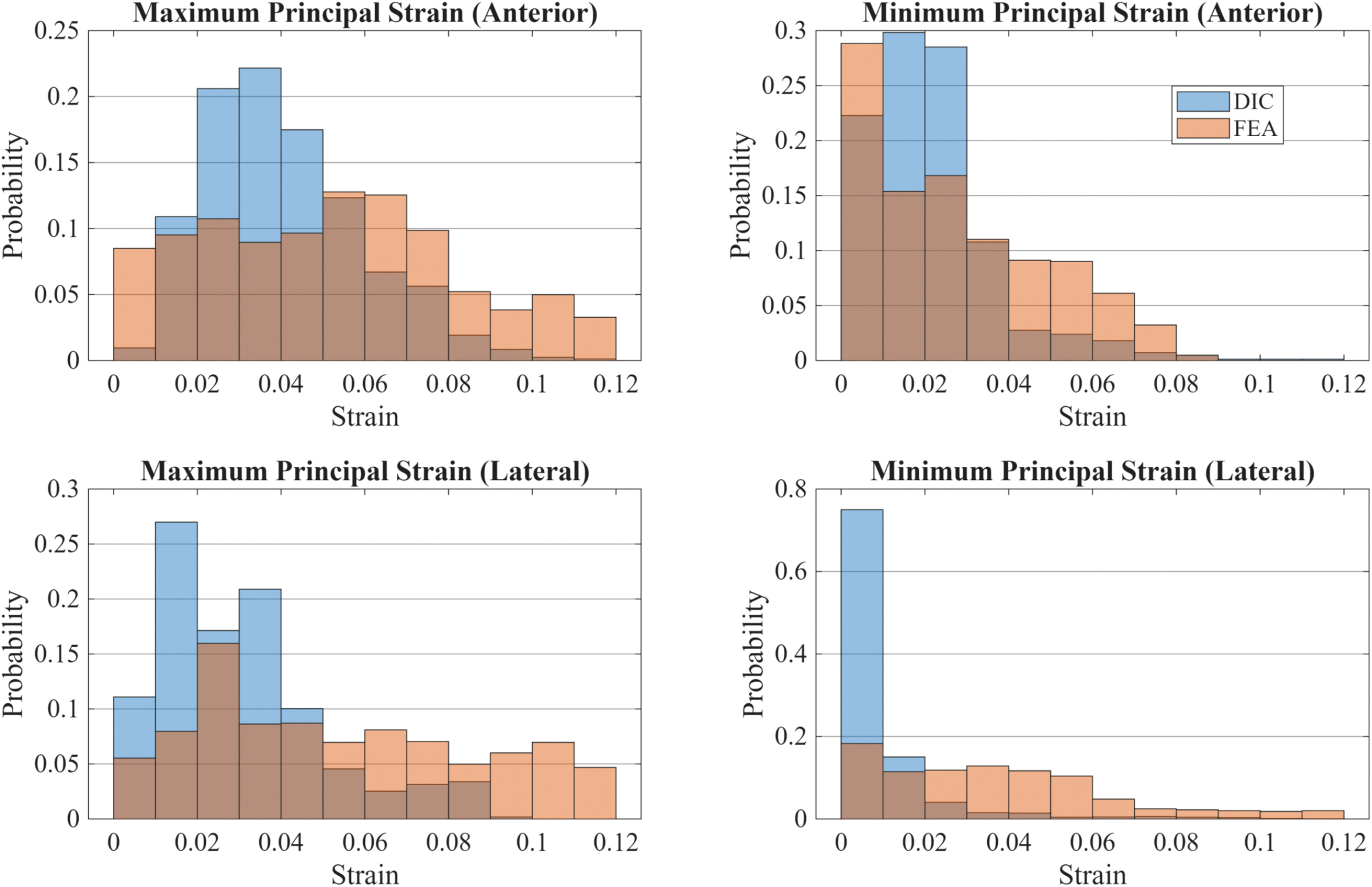
Probability distribution histograms of DIC and FEA measurements of principal strains on the liner surface during the vertical loading task when the vertical force was 810 N and the anteroposterior force was 50 N.

Maximum and minimum principal strains during the vertical loading task were compared between DIC (Fig 7) and FEA (Fig 8). Maximum and minimum principal strains represent tension and compression on the liner surface, respectively. At the time point of interest, the vertical ground reaction force was 0.96 times body weight and the anteroposterior ground reaction force was 0.06 times body weight (directed posteriorly). Within the anterior aspect of the measurable strain region, mean (SD) maximum principal strain was 0.041 (0.018) and 0.051 (0.030) for DIC and FEA, respectively, and mean (SD) minimum principal strain was 0.021 (0.016) and 0.027 (0.022) for DIC and FEA, respectively. The BC was 0.91 and 0.94 for maximum and minimum principal strain, respectively, indicating excellent DIC-FEA agreement (Fig 9). Within the lateral aspect of the measurable strain region, mean (SD) maximum principal strain was 0.030 (0.020) and 0.061 (0.041) for DIC and FEA, respectively, and mean (SD) minimum principal strain was 0.010 (0.016) and 0.045 (0.039) for DIC and FEA, respectively. The BC was 0.81 and 0.73 for maximum and minimum principal strain, respectively, indicating moderate to good DIC-FEA agreement, lower than that observed in the anterior aspect (Fig 9).

**Fig 10:**
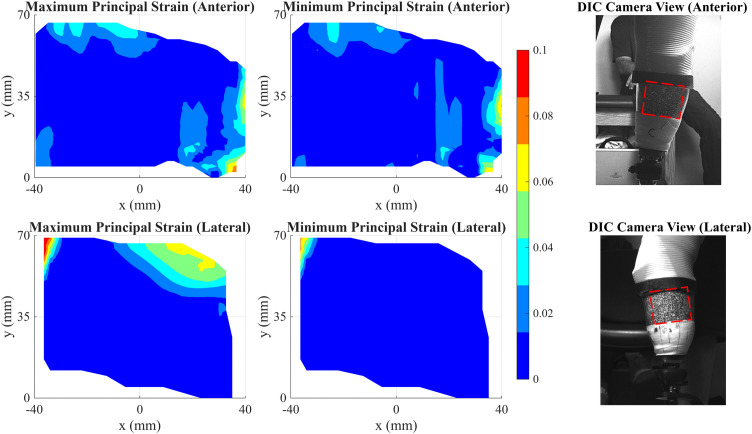
DIC measurements of principal strains on the liner surface when the vertical force was 780 N and the anteroposterior force was 130 N during the half-step loading task.

**Fig 11:**
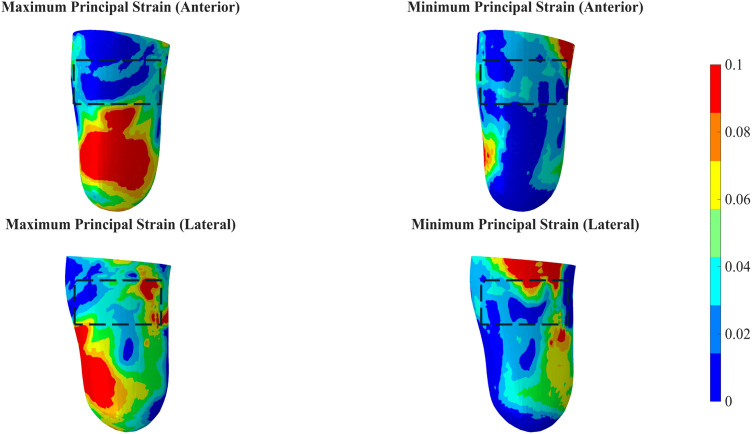
FEA measurements of principal strains on the liner surface when the vertical force was 760 N and the anteroposterior force was 160 N during the half-step loading task. Black box indicates the approximate location of DIC measurable strain region.

**Fig 12:**
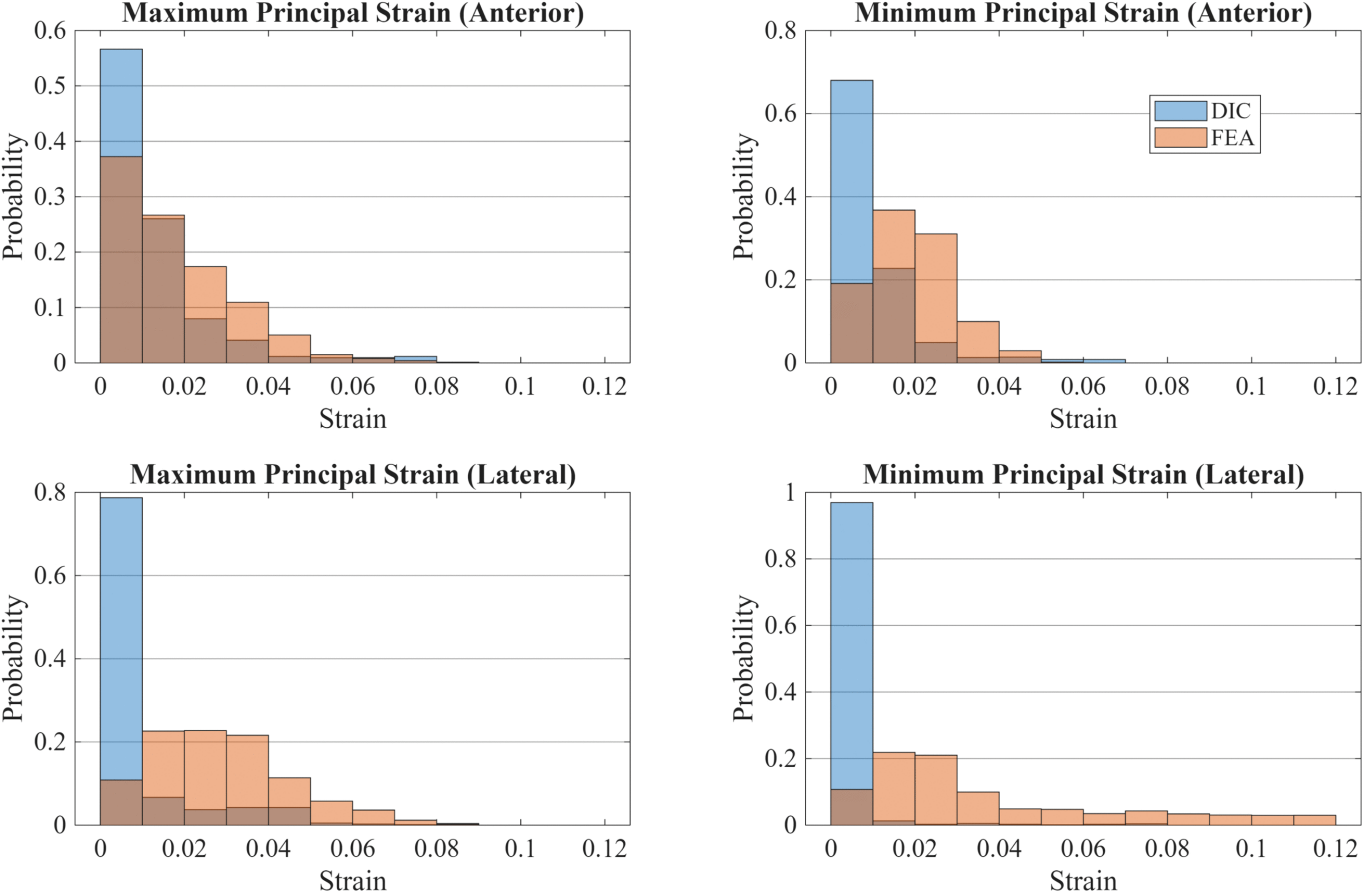
Probability distribution histograms of DIC and FEA measurements of principal strains on the liner surface during the half-step loading task when the vertical force was 760 N and the anteroposterior force was 160 N.

Maximum and minimum principal strains during the half-step loading task were compared between DIC (Fig 10) and FEA (Fig 11). At the time point of interest, the vertical ground reaction force was 0.92 times body weight, the anteroposterior ground reaction force was 0.15 times body weight (directed posteriorly), and the knee was fully extended. Within the anterior aspect of the measurable strain region, mean (SD) maximum principal strain was 0.013 (0.013) and 0.017 (0.015) for DIC and FEA, respectively, and mean (SD) minimum principal strain was 0.009 (0.010) and 0.018 (0.010) for DIC and FEA, respectively. The BC was 0.96 and 0.83 for maximum and minimum principal strain, respectively, indicating good to excellent DIC-FEA agreement (Fig 12). Within the lateral aspect of the measurable strain region, mean (SD) maximum principal strain was 0.009 (0.012) and 0.028 (0.016) for DIC and FEA, respectively, and mean (SD) minimum principal strain was 0.007 (0.004) and 0.044 (0.037) for DIC and FEA, respectively. The BC was 0.71 and 0.46 for maximum and minimum principal strain, respectively, indicating poor to moderate DIC-FEA agreement, lower than that observed in the anterior aspect (Fig 12).

## Discussion

The goals of this single-participant, feasibility study were to demonstrate the use of DIC to measure prosthesis liner strains of a unilateral transtibial prosthesis user during two stepping tasks and conduct a preliminary comparison of these strains with those predicted from an FEA model. This is the first study that we are aware of that attempted to evaluate strains on a residual limb within a prosthetic socket during ecologically valid loading conditions. DIC-based measurements of liner strain were on the order of 0.0–0.1 when the limb was exposed to ground reaction forces near body weight during two stepping tasks. Strains were also shown to be sensitive to loading conditions between the two tasks and likely to the bony anatomy, as discussed below. A challenge with developing FEA models of a human limb for prosthesis-related applications is determining the mechanical properties of the tissues. Indeed, this has resulted in researchers using a range of values [11,25]. As shown here, the direct measurement of strains using DIC can assist with this process and can provide a level of face validity not typically achieved by FEA alone.

While we are not aware of any studies reporting DIC liner strain data obtained under similar conditions for comparison, Solav et al. [22] used DIC to determine skin strains of the residual limb of an individual with a unilateral transtibial amputation. While sitting with the residual limb bare and unsupported, peak strains were ~0.45 in compression and ~0.1 in tension during knee flexion and extension. While this task differs significantly from that investigated here, the order of magnitude of these strains is the same as the liner strains measured here. Differences of strain magnitude measured by Solav et al. [22] and our study could stem from several factors including the different measurement surfaces (bare skin vs. liner exterior), constraining effects of the socket, different loading conditions, and participant differences considering that both studies included only a single participant. Previous FEA studies of transtibial residual limbs have mainly reported internal soft tissue strains which differ based on loading tasks and modelling assumptions. For example, Portnoy et al. [33] reported peak compressive strains of ~0.85 and shear strains of ~1.06 in the internal soft tissues at the distal tibia during a single-leg quiet standing task. However, Mbithi et al. [29] reported peak compressive strains of ~0.41–0.81 and shear strains of ~0.06–0.15 in the same region during walking. Compressive strains in the internal soft tissue at distal tibia have also been shown to depend on modelling parameters, especially soft tissue stiffness, and could vary from ~0.2 to ~0.8 [34]. Such variabilities in strain values underscore the importance of experimental measurements for validating model predictions.

DIC showed higher localized maximum principal liner strains on the anterior aspect of the limb that may be associated with the tibial tuberosity and on the lateral aspect of the limb that may be associated with the fibular head. This may indicate higher contact pressure and shear stresses on the skin at these regions. Previous FEA studies have shown that peak pressure, contact pressure, and shear stress on the residual limb skin were observed at proximal tibia [14,30,35]. The direct measurement of liner strains using DIC provides a level of face validity not achieved by FEA alone.

A significant difference in liner strain pattern was identified between the two tasks. Compared to the vertical loading task (top left in Figs 7 and 8), both DIC (top left in Fig 10) and FEA (top left in Fig 11) showed liner strains on the anterior aspect of the tibia to be lower proximally and higher distally during the half-step task. This may be attributed to the anteroposterior component of the ground reaction force during the half-step task that would tend to rotate the prosthesis/socket relative to the residual limb. That both DIC and FEA exhibited these differences provides additional face validity and agreement of these two methods.

FEA liner strain fields (Figs 8 and 11) exhibited better general agreement with DIC (Figs 7 and 10) in maximum principal strain compared to minimum principal strain. This may be related to modelling soft tissues as linear elastic rather than hyperelastic. Hyperelastic models can better reproduce the compressive behavior of soft tissues [36]. Hyperelastic models were initially evaluated in our FEA model, but they either caused convergence instability in the solver, a limitation also reported by Cagle et al. [37] in their comparable transtibial FEA model, or caused larger disagreement with DIC measurements as compared to the linear elastic properties. FEA also showed better agreement with DIC in the anterior aspect compared to the lateral aspect of the residual limb. The lateral discrepancy was more pronounced during the half-step loading task, likely due to the omission of frontal and transverse plane moments in the FEA model; these moments are present during this task and could influence lateral strain distribution.

This study had multiple limitations and opportunities for improvement that are worth noting. Despite these, we believe the preliminary work reported here will be beneficial to the research community and contribute to future efforts with similar goals. First, this study was conducted on a single participant, which limits the generalizability of the findings. The results should be interpreted as preliminary and indicative of feasibility. Second, we acknowledge that our methodology first selected FEA model soft tissue material properties that minimized the differences in strains between DIC and FEA and then compared these same strains to assess the face validity of FEA predictions, which is best interpreted as a calibration rather than an independent validation. However, disassociating material property selection from FEA model assessment may provide a more rigorous validation process. Third, the soft tissue mechanical properties used within the FEA model were determined using a crude trial-and-error process and likely would benefit from a more rigorous mathematical [20] or sophisticated optimization process that was beyond the scope of this preliminary study. Fourth, the region over which DIC was able to determine strain was limited in size, representing approximately 10% of the total liner surface area. The boundaries of our DIC region were limited distally by the wrapping used to maintain the structural integrity of the socket-pylon connection, proximally by a sealing sleeve needed for prosthesis suspension, and circumferentially by only having access to two DIC cameras. FEA strain results were extracted exclusively from the region corresponding to the DIC field of view rather than from the full liner surface. Additionally, the inability to precisely locate the DIC region on the FEA model introduces uncertainty in region correspondence between the two methods. DIC-FEA comparisons and claims of agreement were therefore limited to the measured region, and regions of high strain outside of the measured region may have been missed. Future studies with a larger DIC region and more precise spatial registration between DIC and FEA will facilitate comparisons. Fifth, bone anatomy in the FEA model was based on a standardized open-access model and tape measurements on the residual limb. This may have resulted in discrepancies in bony anatomy geometry and liner strain differences between DIC and FEA. Sixth, the application of optical-based DIC limited our analysis to the liner and not the skin or other tissues of the limb that are accessible using FEA and likely more closely related to socket comfort. Seventh, the diagnostic socket used here was a total surface bearing socket that has material properties different from those of commercial sockets. Results may therefore differ with sockets using a different fitting strategy or with commercial sockets. Eighth, frontal and transverse plane moments were neglected, and the external loading applied to the limb was determined assuming static equilibrium. Inertial effects were also neglected, which is justified by the quasi-static nature of the tasks. Additionally, the center of pressure was assumed to be aligned with the tibial axis. Neglecting frontal and transverse plane moments may introduce bias in lateral strain estimates, while the assumed center of pressure alignment may introduce asymmetric bias in anterior–posterior strain distributions across the liner surface. Future work incorporating full six-degree-of-freedom loading would enable simulation of more ecologically valid conditions. Ninth, to minimize friction between the paint used to apply the speckled pattern on the liner and the interior of the socket, a water-based lubricant was applied to the interior of the socket prior to donning. This lubricant is not typically used by prosthesis users in practice and caused a frictionless liner-socket contact. Tenth, real prosthetic liners vary locally in thickness and stiffness; however, for simplicity, the liner was modeled as a uniform shell with constant thickness and stiffness. Eleventh, glare from our light sources reflecting off the clear diagnostic liner was a challenge which we attempted to minimize by using soft light sources and proper positioning of these relative to the testing area, but could not be entirely eliminated due to the movements of the socket during testing. Lastly, glare issues were more manageable under controlled, quasi-static loading conditions rather than dynamic gait. Therefore, as a preliminary study, simple controlled tasks were intentionally selected before progressing towards more ecologically valid loading conditions in the future.

In conclusion, this is the first study that we are aware of that attempted to evaluate strains on a residual limb within a prosthetic socket under somewhat realistic gait loading conditions. Maximum and minimum principal strains showed generally comparable distributions between DIC and FEA, especially in the anterior aspect of the residual limb. Although these preliminary results were only for a single participant, they support future investigation of DIC for informing the development of FEA models for prosthetic applications.

## Supporting information

S1 Table FEA strain results for tested Young’s modulus values. (PDF)

S2 File Mesh convergence analysis. (PDF)
