# Supplementary material for: A preliminary comparison of prosthetic socket liner strain determined using digital image correlation and finite element analysis
Source: PLoS One. 2026 Jul 14;21(7):e0353881. doi: 10.1371/journal.pone.0353881 (PMC13367698; doi:10.1371/journal.pone.0353881)
Supplement: S1 Table — (PDF) [file pone.0353881.s001.pdf]

Table 1. Mean and standard deviation of maximum and minimum principal strains on the liner surface for DIC measurements and FEA models with varying Young's modulus values, and Bhattacharyya coefficient of agreement between DIC and each FEA model strain probability histograms, during the vertical loading task. [a]

Method	Anterior		Lateral	
	Max Principal	Min Principal	Max Principal	Min Principal
FEA (E = 0.1 MPa)	0.104 (0.062), 0.67	0.048 (0.039), 0.83	0.102 (0.70), 0.64	0.068 (0.061), 0.59
FEA (E = 0.2 MPa)	0.069 (0.039), 0.81	0.032 (0.027), 0.92	0.079 (0.052), 0.74	0.064 (0.067), 0.65
FEA (E = 0.3 MPa)	0.051 (0.030), 0.91	0.027 (0.022), 0.94	0.061 (0.041), 0.81	0.045 (0.039), 0.73
DIC	0.041 (0.018)	0.021 (0.016)	0.030 (0.020)	0.010 (0.016)

Cell values are reported as: mean (SD), BC

[a] Vertical and anteroposterior ground reaction forces at the time point of interest were 810 N and 50 N, respectively.
