# Supplementary material for: A preliminary comparison of prosthetic socket liner strain determined using digital image correlation and finite element analysis
Source: PLoS One. 2026 Jul 14;21(7):e0353881. doi: 10.1371/journal.pone.0353881 (PMC13367698; doi:10.1371/journal.pone.0353881)
Supplement: S2 File — (PDF) [file pone.0353881.s002.pdf]

To establish mesh convergence for the liner, a convergence study was performed by systematically reducing the element seed size. Convergence was defined as less than 5% change in peak maximum principal strain between successive mesh refinements. A liner element size of 2.5 mm satisfied this criterion, resulting in 11,168 liner elements. The FEA model included 89,832 elements in total. Simulation runtime was approximately 200 minutes.

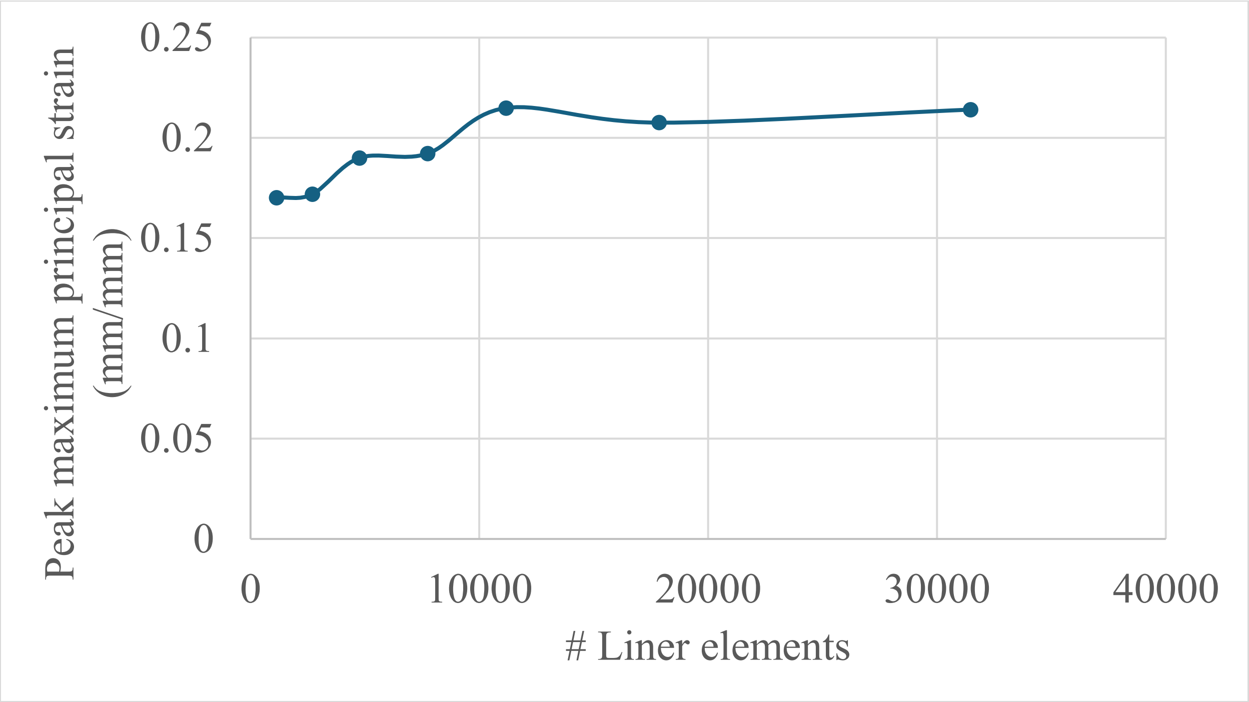

Figure 1. Mesh convergence results for liner. Peak maximum principal strain was calculated over the entire liner surface and when the limb was under vertical loading task.
