# Supplementary material for: A preliminary comparison of prosthetic socket liner strain determined using digital image correlation and finite element analysis
Source: PLoS One. 2026 Jul 14;21(7):e0353881. doi: 10.1371/journal.pone.0353881 (PMC13367698; doi:10.1371/journal.pone.0353881)
Supplement: Attachment — Submitted filename: Response to reviewers.docx [file pone.0353881.s004.docx]

We would like to thank the editor and reviewers for their thoughtful review and constructive comments on our manuscript. We believe that the process of addressing these comments has improved the quality of our manuscript. Our point-by-point responses are provided below in italics and are indicated by >>>. Changes in the manuscript have been highlighted as requested by the journal.

Journal Requirements:

When submitting your revision, we need you to address these additional requirements.

1. Please ensure that your manuscript meets PLOS ONE's style requirements, including those for file naming. The PLOS ONE style templates can be found at

https://journals.plos.org/plosone/s/file?id=wjVg/PLOSOne_formatting_sample_main_body.pdf and

<https://journals.plos.org/plosone/s/file?id=ba62/PLOSOne_formatting_sample_title_authors_affiliations.pdf>.

>>> Thank you for sharing these guidelines. We updated our manuscript to adhere to the PLOS ONE style requirements.

2. Please note that PLOS One has specific guidelines on code sharing for submissions in which author-generated code underpins the findings in the manuscript. In these cases, we expect all author-generated code to be made available without restrictions upon publication of the work. Please review our guidelines at https://journals.plos.org/plosone/s/materials-andsoftware-sharing#loc-sharing-code and ensure that your code is shared in a way that follows best practice and facilitates reproducibility and reuse.

>>> Our model input file and data points have been stored in Virginia Tech research repository:

https://doi.org/10.7294/32337195

3. Thank you for stating in your Funding Statement:

“This study was supported by the U.S. Army Medical Research and Development Command under award number: W81XWH-21-1-0220. “

Please provide an amended statement that declares *all* the funding or sources of support (whether external or internal to your organization) received during this study, as detailed online in our guide for authors at http://journals.plos.org/plosone/s/submit-now. Please also include the statement “There was no additional external funding received for this study.” in your updated Funding Statement. Please include your amended Funding Statement within your cover letter. We will change the online submission form on your behalf.

>>> We updated our cover letter.

4. Thank you for stating the following financial disclosure: “This study was supported by the U.S. Army Medical Research and Development Command under award number: W81XWH-21-1-0220. “ Please state what role the funders took in the study. If the funders had no role, please state: "The funders had no role in study design, data collection and analysis, decision to publish, or preparation of the manuscript." If this statement is not correct you must amend it as needed. Please include this amended Role of Funder statement in your cover letter; we will change the online submission form on your behalf.

>>> We updated our cover letter.

5. We note that your Data Availability Statement is currently as follows: [All relevant data are within the manuscript.] Please confirm at this time whether or not your submission contains all raw data required to replicate the results of your study. Authors must share the “minimal data set” for their submission. PLOS defines the minimal data set to consist of the data required to replicate all study findings reported in the article, as well as related metadata and methods (https://journals.plos.org/plosone/s/data-availability#loc-minimal-data-set-definition). For example, authors should submit the following data: - The values behind the means, standard deviations and other measures reported; - The values used to build graphs; - The points extracted from images for analysis. Authors do not need to submit their entire data set if only a portion of the data was used in the reported study. If your submission does not contain these data, please either upload them as Supporting Information files or deposit them to a stable, public repository and provide us with the relevant URLs, DOIs, or accession numbers. For a list of recommended repositories, please see https://journals.plos.org/plosone/s/recommended-repositories. If there are ethical or legal restrictions on sharing a de-identified data set, please explain them in detail (e.g., data contain potentially sensitive information, data are owned by a third-party organization, etc.) and who has imposed them (e.g., an ethics committee). Please also provide contact information for a data access committee, ethics committee, or other institutional body to which data requests may be sent. If data are owned by a third party, please indicate how others may request data access.

>>> Our model input file and data points have been stored in Virginia Tech research repository:

https://doi.org/10.7294/32337195

6. If the reviewer comments include a recommendation to cite specific previously published works, please review and evaluate these publications to determine whether they are relevant and should be cited. There is no requirement to cite these works unless the editor has indicated otherwise. [Note: HTML markup is below. Please do not edit.]

Review Comments to the Author:

Reviewer #1: The manuscript presents a novel and valuable study that compares digital image correlation (DIC) and finite element analysis (FEA) for measuring strain in prosthetic liners. The work is well motivated, methodologically detailed, and demonstrates promising agreement between experimental and computational strain estimates. However, the reviewer identifies several important limitations requiring major revision. Key Strengths First known attempt to measure liner strain in vivo under realistic loading conditions. Strong methodological transparency in DIC setup, socket design, and FEA workflow. Good numerical agreement between DIC and FEA strain patterns, supporting the feasibility of this validation approach.

>>> Thank you for your thorough and constructive review. We appreciate the recognition of the novelty and methodological transparency of our work, and we have addressed each of the concerns and comments raised below.

Major Concerns:

•Single participant, limiting generalizability.

>>>Thank you for raising this concern. We agree that the single-participant design limits generalizability. We have revised the manuscript to explicitly acknowledge this limitation and to clarify that this study is a preliminary feasibility investigation.

•Simplified soft‑tissue modelling (linear elastic instead of hyperelastic).

>>> We agree that modeling soft tissues as linear elastic is a simplification that does not fully capture their nonlinear behavior. Hyperelastic models were explored; however, they resulted in numerical instabilities under the applied loading conditions and the model did not converge. This issue with hyperelastic models was also reported by Cagle et al. (2018). Due to the focus of this study on feasibility and our limited resources in quantifying subject-specific soft tissue behavior, linear elastic properties were used for modelling soft tissue. We have noted this in the manuscript in the fifth paragraph of the Discussion.

Reference:

“Cagle JC, Reinhall PG, Allyn KJ, McLean J, Hinrichs P, Hafner BJ, et al. A finite element model to assess transtibial prosthetic sockets with elastomeric liners. Medical and Biological Engineering and Computing. 2018;56(7):1227–40.”

Suggested improvement:

Lines 19–37 •Comment: The abstract is clear and well structured, but it should acknowledge the single participant design explicitly, as this strongly limits generalizability. •Suggestion: Add 1–2 sentences describing key limitations upfront to set realistic expectations.

>>> We agree that the single-participant design is an important limitation that should be mentioned upfront. We have revised the abstract to acknowledge this limitation and that the findings are preliminary.

•Lines 49–58: You highlight variability in FEA stress predictions due to a lack of validation. This is an excellent justification. However, more explanation of typical error magnitudes or clinical relevance thresholds would strengthen the rationale.

>>> We have added a sentence to this section noting that FEA stress predictions have been experimentally validated using pressure sensors by other studies, with errors of up to 12 KPa, and that such errors are clinically significant given that repetitive interface stresses as low as 4–23 kPa have been associated with skin damage risk in prosthesis user.(Second paragraph of the Introduction)

•Line 60: DIC is presented as widely accepted; consider citing more prosthetics-specific DIC applications to position the study within the field.

>>> To be clear, DIC is widely accepted in engineering mechanics, but has not been used extensively in prosthesis-related work. In response to the reviewer’s comment, we have made this more clear and included an additional DIC study about prosthetic sockets by Saey et al. (2019). (Third paragraph of the Introduction)

•Lines 74–81: The two research goals are clearly stated. However, consider emphasising that this is an exploratory single subject feasibility study, not a validation study.

>>>We have revised the statement of the goals to explicitly indicate the single-participant and feasibility nature of the study. (Fourth paragraph of the Introduction)

•Lines 85–89: Single participant; demographic justification is absent. Authors should state why this individual was chosen (e.g., typical limb geometry, availability).

>>> The participant was selected based on a) having a below knee amputation at least one year prior; b) having a well-fitting prosthesis that was used daily; and c) schedule availability. We have added this to the paragraph.

•Line 100: Speckle pattern creation using fabric dye and thickener requires justification—does this alter liner compliance or friction properties?

>>> The speckled liner has a rougher surface than normal liner and this will increase the friction coefficient. For this reason, we decided to apply lubricant to remove this effect and have a frictionless liner-socket interface. We have discussed this as a potential limitation in the sixth paragraph of Discussion.

•Lines 105–118: The two tasks (vertical loading, half step) are simple and controlled. However, they do not reflect everyday gait dynamics. Authors should justify why a more realistic gait was excluded.

>>> We agree that selected tasks do not represent full gait dynamics. Full gait was excluded because glare and reflections on the transparent socket were more manageable under-controlled and quasi-static conditions than dynamic walking. Additionally, as a preliminary study, we focused on simple controlled tasks to establish a methodology prior to advancing to more ecologically valid conditions in future work. We have discussed this as a potential limitation in the sixth paragraph of Discussion.

•Lines 118–122: Because only two DIC cameras were available, separate trials were required for anterior and lateral views. This creates potential alignment inconsistencies between datasets and should be acknowledged earlier.

>>> We agree that separate trials for anterior and lateral views could introduce inconsistencies across trials. To minimize the inconsistencies, the participant was provided with clear verbal instructions and visual markers on the forceplate for prosthetic foot positioning. We have added this explanation to the manuscript. We have added this in the third paragraph of Methods.

•Lines 137–140: More detail is needed on the DIC reconstruction error, stereocalibration accuracy, and noise filtering.

>>We have included more information about DIC calibration and filters in the Digital image correlation section.

•Lines 140–145: The strain measurement region was extremely limited. This methodological constraint strongly weakens the validity of comparing full field FEA results with partial-field DIC data.

>>> The measurable DIC region is limited due to proximal sleeve and distal wrap for maintaining the structural integrity of the socket. It is also worth noting that the approximate location of the corresponding region has been shown on FEA results, and all the comparisons were limited to only the corresponding region on the FEA. We acknowledge, however, that this approach limits the ability to draw conclusions about strain distributions across the full liner surface. This issue has been acknowledged as one of the study limitations (Sixth paragraph of the Discussion)

•Lines 155–157: Bone anatomy derived from an open-source model and scaled using tape measurements introduces significant geometric uncertainty. Consider quantifying potential anatomical mismatch.

>>> We agree that our method in bone sizes and positioning could introduce uncertainty. Formal quantification of anatomical mismatch was not possible for us due to lack of imaging data from the participant residual limb bones. We have acknowledged this as a limitation of our study. (Sixth paragraph of the Discussion)

•Lines 166–170: Soft tissues modelled as linear elastic contradict well-established nonlinear, viscoelastic behaviour. A more rigorous rationale is needed.

>>> We agree that linear elastic does not represent the full nonlinear behavior of soft tissues and have acknowledged this as a limitation. Hyperelastic material models were evaluated but could not be used due to solver convergence instability in the FEA software. Cagle et al. (2018) also reported the model convergence instability with hyperelastic models of soft tissues and alternatively adopted a linear elastic model. We have provided more justification in our discussion. (Fifth paragraph of the Discussion)

•Lines 183–190: The loading simplifications (no frontal/transverse plane moments, assumed COP alignment, no inertial effects) reduce ecological validity. Authors should evaluate how these simplifications might bias results.

>>> Neglecting frontal/transverse plane moments could introduce bias of strain values, especially in the lateral side of the residual limb. These moments were neglected following the approach used in prior transtibial prosthetic FEA studies (Cagle et al. (2018) & Mbithi et al. (2022)). Assuming the center of pressure to be aligned with the tibial axis could introduce error in asymmetric strain distribution. Regarding inertial effects, the two loading tasks were intentionally selected quasi-static tasks that were performed slowly to minimize inertial effects, which is consistent with the feasibility scope of this study. We have added more information regarding these limitations to the manuscript. (Sixth paragraph of the Discussion)

•Lines 200–212: The agreement between DIC and FEA strains is well presented. However, statistical error metrics (e.g., RMSE, correlation, Bland–Altman plots) are missing.

>>> Due to differences in mesh discretization between FEA and DIC strain fields, point-wise comparison was not feasible. Gomez et al. (2018) proposed a methodology for such comparisons by comparing strain distribution histograms. In response to this comment, we have adapted their approach and generated probability distribution histograms of principal strains for both FEA and DIC and quantified their overlap using the Bhattacharyya coefficient (BC), a measure of overlap between two probability distributions where a value of 1 indicates complete overlap and 0 indicates no overlap. We additionally compared mean and standard deviation of principal strains between FEA and DIC. Accordingly, we have added a section to our Methods title as “DIC and FEA comparison” and updated our Results to report these comparison measures.

Reference:

Gomez AD, Stone ML, Bayly PV, Prince JL. Quantifying Tensor Field Similarity With Global Distributions and Optimal Transport. Med Image Comput Comput Assist Interv. 2018;11071:428–36.

•Lines 211–218: For the half-step task, a substantial discrepancy appears in the lateral minimum and maximum strains. Authors should explain this more clearly rather than briefly noting it.

>>> We agree that the lateral strain discrepancy during the half-step task needs more explanation. The higher lateral discrepancy between FEA and DIC during the half-step task could be due to the omission of frontal and transverse plane moments in the FEA model, which are present during this task and particularly relevant to lateral strain distribution. We have included a discussion of this discrepancy. (Fifth paragraph of the Discussion)

•Figures: Clear, but inconsistent colour scaling between DIC and FEA maps makes direct visual comparison difficult.

>>> Upon review, the color scaling for DIC results (Figures 7 and 10) and FEA results (Figures 8 and 11) appear identical in our original versions of these figures. To the reviewer’s point, this was our intent. Perhaps the color mismatch mentioned by both reviewers was an artifact of the uploading and pdf construction process. We will take a closer look at this after the PLOS ONE submission system compiles a final pdf and before submitting the manuscript.

•Lines 248–263: The authors compare their strain results to prior DIC studies. Suggest adding discussion on why magnitude differences occur (e.g., liner vs skin measurements).

>>> We have included more discussion regarding potential contributors to strain differences. Different load and boundary conditions and the single-participant nature of these studies could cause strain differences. There is a need for future studies to simultaneously measure skin and liner strains with more participants. (Second paragraph of the Discussion)

•Lines 273–278: The explanation of task sensitivity is insightful; however, the discussion should address how the simplified load case compares to actual gait loading.

>>> We have added discussion of a limitation that simplifications in the load cases might bias the strain magnitudes that would be observed during full gait. Future studies incorporating full gait loading will be needed to characterize liner strain distributions under more ecologically valid conditions. (Sixth paragraph of the Discussion)

•Lines 282–285: The authors acknowledge the limitation of linear tissue modelling, but the dismissal of hyperelastic models requires more detail — what specific instabilities occurred?

>>> Hyperelastic models were evaluated but could not be used due to solver convergence instability in the FEA software. Cagle et al. (2018) also reported the model convergence instability with hyperelastic models of soft tissues and alternatively adopted a linear elastic model. We have provided more justification in our discussion. (Fifth paragraph of the Discussion)

•Lines 288–316

1.Repeatability: No intra-trial variability analysis or repeated measures.

>>> We acknowledge that intra-trial variability was not assessed in this study and agree it is important to do so in future work.

2.Liner nonuniformity: Real prosthetic liners vary locally in thickness and stiffness, unlike the modelled uniform shell.

>>> The liner was modeled as a uniform shell for simplicity with constant thickness and stiffness, and its thickness was set to the measured liner thickness value at the DIC measurement region. Liner stiffness was based on material properties reported for the specific liner product used in the study by:“Cagle et al. (2018). Characterization of prosthetic liner products for people with transtibial amputation. JPO: Journal of Prosthetics and Orthotics, 30(4), 187–199.” (Sixth paragraph of the Discussion)

3.Effect of lubricant and clear socket: The test conditions differ materially from real-world prosthesis use.

>>> We agree that both the transparent socket and lubricant are different from real-world, and have acknowledged them as limitations. (Sixth paragraph of the Discussion)

4.Region mismatch: The limited DIC area may not correspond perfectly to the FEA region assessed.

>>> We have included this in our limitations. (Sixth paragraph of the Discussion)

•Strong concluding summary; however, avoid overgeneralizing given the single participant nature.

•Suggest softening claims about “good agreement,” emphasising that findings are preliminary.

>>> We have revised the conclusion section and soften our conclusions regarding generalizability.

Reviewer #2: Review Comments to the Author:

The manuscript presents a preliminary comparison of prosthetic liner strains measured using digital image correlation (DIC) and predicted using finite element analysis (FEA) in a unilateral transtibial prosthesis user. The study addresses an important and longstanding challenge in prosthetic biomechanics: experimental validation of FEA models used to estimate interface mechanics. The integration of DIC with subject-specific FEA modeling under load-bearing conditions is novel and technically interesting. Overall, the study demonstrates methodological feasibility and is clearly written. However, several issues related to validation rigor, experimental design, and interpretation must be addressed before the conclusions can be considered adequately supported.

>>> Thank you for your thoughtful and constructive evaluation of our manuscript. We appreciate the recognition of the novelty of our study. We have addressed each of the comments raised below.

Major Comments:

1. Calibration vs. Validation

The manuscript states that three values of Young’s modulus were evaluated and that 0.3 MPa was selected because it provided the closest match to DIC measurements. However, this approach constitutes calibration rather than independent validation. After tuning material properties to match DIC, the manuscript then concludes that FEA and DIC exhibit “good agreement.”

To strengthen the scientific rigor:

• Clearly distinguish between model calibration and model validation.

• Provide quantitative error metrics (e.g., RMSE, mean absolute error, percent difference, spatial correlation).

• Present strain comparisons for all tested material properties to show how sensitive agreement is to stiffness selection.

• Avoid language implying independent validation unless supported by independent data

>>> We have revised our study goals and discussions to avoid implying independent validation. We have also explicitly acknowledged our methodology as calibration. (Sixth paragraph of the Discussion)

*Due to differences in mesh discretization between FEA and DIC strain fields, point-wise quantitative comparison was not feasible. Gomez et al. (2018) proposed a methodology for such comparisons by comparing strain distribution histograms; adapting their approach, we generated probability distribution histograms of principal strains for both FEA and DIC and quantified their overlap using the Bhattacharyya coefficient (BC), a measure of overlap between two probability distributions where a value of 1 indicates complete overlap and 0 indicates no overlap. We additionally compared mean and standard deviation (SD) of principal strains between FEA and DIC. Accordingly, we have added a section to our Methods title as “DIC and FEA comparison” and updated our Results to report these comparison measures.*

*We have provided S1 Table in Supporting information that presents FEA results for the tested soft tissue materials and compares them with DIC measurements.*

*Reference:*

*Gomez AD, Stone ML, Bayly PV, Prince JL. Quantifying Tensor Field Similarity With Global Distributions and Optimal Transport. Med Image Comput Comput Assist Interv. 2018;11071:428–36.*

2. Sample Size and Generalizability

The study includes only one participant. While acceptable for a feasibility study, this limits the strength of conclusions. Anatomical variability, tissue properties, and socket fit vary substantially across individuals.

The manuscript should:

• Explicitly frame the study as a single-subject feasibility or proof-of-concept study.

• Avoid generalizable claims regarding prosthetic socket design.

• Clearly acknowledge that findings may be subject-specific.

>>> We agree that the single-participant design limits the strength of conclusions and requires clear communication throughout the manuscript. We have revised the manuscript to consistently frame this as a single-participant feasibility study, soften conclusions to reflect the preliminary and subject-specific nature of the findings, and acknowledge that results should not be generalized to broader populations.

3. Quantitative Assessment of Agreement

Agreement between DIC and FEA is described largely qualitatively (“good agreement”) and supported by selected peak strain values. However:

• No mesh convergence study is reported for FEA.

• No statistical or spatial comparison metrics are included.

I strongly recommend adding:

• Quantitative comparison metrics.

• A spatial difference map between DIC and FEA strain fields.

• Mesh refinement justification.

>>> As noted above, point-wise mapping between FEA and DIC was not feasible, and we could not conduct spatial comparison between two methods. Alternatively, we compared histograms of strain distribution over the region to compare FEA and DIC results using the methodology reported by Gomez et al. (2018). The revised manuscript now reports mean, standard deviation, and Bhattacharyya coefficients (BC) for both maximum and minimum principal strain distributions within the anterior and lateral measurement regions for both tasks. Moreover, a mesh convergence study was performed and is provided as supporting information (S2 File)

4. Modeling Assumptions and Sensitivity:

The FEA model includes several simplifying assumptions: • Linear elastic soft tissues • Frictionless liner–socket interface • Simplified loading (sagittal plane only) • Assumed center-of-pressure alignment • Non-subject-specific bone geometry

These assumptions may substantially influence strain distribution.

While limitations are acknowledged, the manuscript would benefit from:

• A brief sensitivity analysis (e.g., effect of friction coefficient or stiffness variation on strain magnitude).

• Clarification of how loads were applied (to bone nodes, rigid body, distributed load).

• Discussion of how assumptions may bias agreement with DIC.

>>> Thank you for the suggestions. We have provided sensitivity analysis of soft tissue materials in S1. Table. We assumed the liner-socket contact to be frictionless due to the application of lubricant.

Loads were applied to a reference node on the tibia head, with vertical and anteroposterior forces and the resultant sagittal plane moment derived from ground reaction force measurements. We have added this clarification to the third paragraph of the Finite element analysis section in the Methods.

The modeling assumptions are acknowledged in the Discussion section and how they could cause bias on strain predictions. (Sixth paragraph of the Discussion)

5. Limited DIC Measurement Region

The measurable strain region is approximately 7 cm in height and 8 cm circumferentially. This represents a limited portion of the liner surface.

The manuscript should:

• Clarify what percentage of total liner surface was analyzed.

• Discuss whether high-strain regions outside this window may have been missed.

• Ensure that claims of agreement are limited to the measured region

>>> We have revised our limitations to explicitly state that the DIC measurement region represented approximately 10% of the total liner surface area and to acknowledge that all the comparisons were limited to this region, and high strains out of this region might have been missed. (Sixth paragraph of the Discussion)

Minor Comments:

• Provide additional DIC processing details (subset size, step size, smoothing, calibration error).

>>> We have provided more DIC details and calibration information in the Methods.

• Ensure identical color scales are used when comparing DIC and FEA strain maps.

>>> Upon review, the color scaling for DIC results (Figures 7 and 10) and FEA results (Figures 8 and 11) appear identical in our original versions of these figures. To the reviewer’s point, this was our intent. Perhaps the color mismatch mentioned by both reviewers was an artifact of the uploading and pdf construction process. We will take a closer look at this after the PLOS ONE submission system compiles a final pdf and before submitting the manuscript.

• Soften statements such as “validate FEA model results” to reflect the preliminary nature of the study.

>>> We have revised our manuscript to avoid stating that FEA results were validated in this study. However, the methodology here could also guide future studies for FEA model validation.

• Consider depositing raw DIC data and model inputs in a public repository to align with PLOS data transparency standards.

>>> Thank you for this suggestion. We have provided a repository for our data.

Reviewer #3: The present study demonstrates the use of DIC to measure prosthesis liner strains of a unilateral transtibial prosthesis user and conducts a preliminary comparison of these strains with those predicted from an FEA model.

>>> Thank you for the constructive comments and for highlighting important methodological challenges associated with DIC. We have answered each question as follows:

The use of DIC has certain challenges as compared to the other experimental techniques, such as: 1. Unstable Speckle Pattern-Sweat or skin stretching alters the speckle pattern, Prosthetic socket friction can erase or distort the pattern, Long walking trials degrade pattern quality. It will be useful mentioning how a Stable Speckle pattern was assured for this study.

>>> The speckle pattern remained stable throughout data collection. No visible pattern degradation was observed after trials. The lubricant applied to the interior of the socket prior to donning also reduced the friction and may have helped to prevent the pattern from degradation. Additionally, the quasi-static nature of the two tasks minimized the mechanical demands on the pattern.

2. Secondly Walking involves Dynamic Motion and leads to Motion blurring if cameras are not high speed. Also, Synchronization errors between cameras and gait cycle phases. How are these Issues addressed?

>>>The two DIC cameras operated at 50 frames per second and were hardware-synchronized using a programmable timing unit to eliminate synchronization errors between cameras. The two tasks were quasi-static, which minimized the velocity of limb motion during data collection and reduced the likelihood of motion blur.

3. Another major drawback of using DIC is that for minor changes in prostheses alignment cause large changes in stress distribution, which makes DIC results difficult to generalize. How is the alignment issue handled in the present study?

>>> We do not disagree with the reviewer. We used a licensed and experienced prosthetist to set prosthesis alignment. We have also softened the conclusions and generalizability of our results in the manuscript given that we only tested one participant and thus our results can be dependent upon individual characteristics.

4. For a prostheses user, there is a movement of skin moves relative to the underlying bone and muscle known as soft tissue artifact. This phenomenon is known as soft tissue artifact. How present technique addresses this issue and what is the error percentage for the same.

>>> The goal of this study, and the broader goal of applying DIC to prosthesis applications, is to evaluate liner or skin strain and use that either alone or with FEA estimates of liner, skin, and/or tissue strain to better design comfortable prosthesis with low risk of soft tissue issues. The relative movement between the liner and the underlying bone was not a focus and thus not directly measured in this study. It represents a direction for future work.

Also, there are some studies done in past that uses DIC for Prostheses Socket Pressure Measurement. Refer to the following study -Evaluation of the influence of cyclic loading on a laser sintered transtibial prosthetic socket using Digital Image Correlation (DIC) by Saey et al. Annu Int Conf IEEE Eng Med Biol Soc, 2019

>>> Thank you for suggesting this study. This study has been added to the Introduction to support the growing application of DIC in prosthetics research
