# Supplementary material for: A preliminary comparison of prosthetic socket liner strain determined using digital image correlation and finite element analysis
Source: PLoS One. 2026 Jul 14;21(7):e0353881. doi: 10.1371/journal.pone.0353881 (PMC13367698; doi:10.1371/journal.pone.0353881)
Supplement: Peer review history

# Peer review: Decision Letter 0

**DOI:** 10.1371/journal.pone.0353881.r001

**Authors:** Alessandra Aldieri

22 Apr 2026

PONE-D-26-07309

A Preliminary Comparison of Prosthetic Socket Liner Strain Determined Using Digital Image Correlation and Finite Element Analysis

PLOS One

Dear Dr. Philen,

Thank you for submitting your manuscript to PLOS ONE. After careful consideration, we feel that it has merit but does not fully meet PLOS ONE’s publication criteria as it currently stands. Therefore, we invite you to submit a revised version of the manuscript that addresses the points raised during the review process.

As highlighted by the Reviewers, there a number of major issues concerning the study design and some methodological approaches particularly which need to be addressed prior to publication.

Please submit your revised manuscript by Jun 06 2026 11:59PM. If you will need more time than this to complete your revisions, please reply to this message or contact the journal office at plosone@plos.org. When you're ready to submit your revision, log on to https://www.editorialmanager.com/pone/ and select the 'Submissions Needing Revision' folder to locate your manuscript file.

Please include the following items when submitting your revised manuscript:

- A letter that responds to each point raised by the academic editor and reviewer(s). You should upload this letter as a separate file labeled 'Response to Reviewers'.
- A marked-up copy of your manuscript that highlights changes made to the original version. You should upload this as a separate file labeled 'Revised Manuscript with Track Changes'.
- An unmarked version of your revised paper without tracked changes. You should upload this as a separate file labeled 'Manuscript'.

If you would like to make changes to your financial disclosure, please include your updated statement in your cover letter. Guidelines for resubmitting your figure files are available below the reviewer comments at the end of this letter.

If applicable, we recommend that you deposit your laboratory protocols in protocols.io to enhance the reproducibility of your results. Protocols.io assigns your protocol its own identifier (DOI) so that it can be cited independently in the future. For instructions see: https://journals.plos.org/plosone/s/submission-guidelines#loc-laboratory-protocols. Additionally, PLOS ONE offers an option for publishing peer-reviewed Lab Protocol articles, which describe protocols hosted on protocols.io. Read more information on sharing protocols at https://plos.org/protocols?utm_medium=editorial-email&utm_source=authorletters&utm_campaign=protocols.

As the corresponding author, your ORCID iD is verified in the submission system and will appear in the published article. PLOS supports the use of ORCID, and we encourage all coauthors to register for an ORCID iD and use it as well. Please encourage your coauthors to verify their ORCID iD within the submission system before final acceptance, as unverified ORCID iDs will not appear in the published article. *Only*  the individual author can complete the verification step; PLOS staff *cannot*  verify ORCID iDs on behalf of authors.

We look forward to receiving your revised manuscript.

Kind regards,

Alessandra Aldieri

Academic Editor

PLOS One

Journal Requirements:

When submitting your revision, we need you to address these additional requirements.

1. Please ensure that your manuscript meets PLOS ONE's style requirements, including those for file naming. The PLOS ONE style templates can be found at

https://journals.plos.org/plosone/s/file?id=wjVg/PLOSOne_formatting_sample_main_body.pdf and

https://journals.plos.org/plosone/s/file?id=ba62/PLOSOne_formatting_sample_title_authors_affiliations.pdf.

2. Please note that PLOS One has specific guidelines on code sharing for submissions in which author-generated code underpins the findings in the manuscript. In these cases, we expect all author-generated code to be made available without restrictions upon publication of the work. Please review our guidelines at https://journals.plos.org/plosone/s/materials-and-software-sharing#loc-sharing-code and ensure that your code is shared in a way that follows best practice and facilitates reproducibility and reuse.

3. Thank you for stating in your Funding Statement:

“This study was supported by the U.S. Army Medical Research and Development Command under award number: W81XWH-21-1-0220. “

Please provide an amended statement that declares *all* the funding or sources of support (whether external or internal to your organization) received during this study, as detailed online in our guide for authors at http://journals.plos.org/plosone/s/submit-now.  Please also include the statement “There was no additional external funding received for this study.” in your updated Funding Statement.

Please include your amended Funding Statement within your cover letter. We will change the online submission form on your behalf.

4. Thank you for stating the following financial disclosure:

“This study was supported by the U.S. Army Medical Research and Development Command under award number: W81XWH-21-1-0220. “

Please state what role the funders took in the study.  If the funders had no role, please state: "The funders had no role in study design, data collection and analysis, decision to publish, or preparation of the manuscript."

If this statement is not correct you must amend it as needed.

Please include this amended Role of Funder statement in your cover letter; we will change the online submission form on your behalf.

5. We note that your Data Availability Statement is currently as follows: [All relevant data are within the manuscript. ]

Please confirm at this time whether or not your submission contains all raw data required to replicate the results of your study. Authors must share the “minimal data set” for their submission. PLOS defines the minimal data set to consist of the data required to replicate all study findings reported in the article, as well as related metadata and methods (https://journals.plos.org/plosone/s/data-availability#loc-minimal-data-set-definition).

For example, authors should submit the following data:

- The values behind the means, standard deviations and other measures reported;

- The values used to build graphs;

- The points extracted from images for analysis.

Authors do not need to submit their entire data set if only a portion of the data was used in the reported study.

If your submission does not contain these data, please either upload them as Supporting Information files or deposit them to a stable, public repository and provide us with the relevant URLs, DOIs, or accession numbers. For a list of recommended repositories, please see https://journals.plos.org/plosone/s/recommended-repositories.

If there are ethical or legal restrictions on sharing a de-identified data set, please explain them in detail (e.g., data contain potentially sensitive information, data are owned by a third-party organization, etc.) and who has imposed them (e.g., an ethics committee). Please also provide contact information for a data access committee, ethics committee, or other institutional body to which data requests may be sent. If data are owned by a third party, please indicate how others may request data access.

6. If the reviewer comments include a recommendation to cite specific previously published works, please review and evaluate these publications to determine whether they are relevant and should be cited. There is no requirement to cite these works unless the editor has indicated otherwise.

[Note: HTML markup is below. Please do not edit.]

Reviewers' comments:

Reviewer's Responses to Questions

**Comments to the Author**

1. Is the manuscript technically sound, and do the data support the conclusions?

The manuscript must describe a technically sound piece of scientific research with data that supports the conclusions. Experiments must have been conducted rigorously, with appropriate controls, replication, and sample sizes. The conclusions must be drawn appropriately based on the data presented.

Reviewer #1: Yes

Reviewer #2: Partly

Reviewer #3: Partly

**********

2. Has the statistical analysis been performed appropriately and rigorously?

Reviewer #1: Yes

Reviewer #2: No

Reviewer #3: No

**********

3. Have the authors made all data underlying the findings in their manuscript fully available?

The PLOS Data policy requires authors to make all data underlying the findings described in their manuscript fully available without restriction, with rare exception (please refer to the Data Availability Statement in the manuscript PDF file). The data should be provided as part of the manuscript or its supporting information, or deposited to a public repository. For example, in addition to summary statistics, the data points behind means, medians and variance measures should be available. If there are restrictions on publicly sharing data—e.g. participant privacy or use of data from a third party—those must be specified.

Reviewer #1: Yes

Reviewer #2: Yes

Reviewer #3: Yes

**********

4. Is the manuscript presented in an intelligible fashion and written in standard English?

PLOS ONE does not copyedit accepted manuscripts, so the language in submitted articles must be clear, correct, and unambiguous. Any typographical or grammatical errors should be corrected at revision, so please note any specific errors here.

Reviewer #1: Yes

Reviewer #2: Yes

Reviewer #3: Yes

**********

5. Review Comments to the Author

Please use the space provided to explain your answers to the questions above. You may also include additional comments for the author, including concerns about dual publication, research ethics, or publication ethics. (Please upload your review as an attachment if it exceeds 20,000 characters)

Reviewer #1: The manuscript presents a novel and valuable study that compares digital image correlation (DIC) and finite element analysis (FEA) for measuring strain in prosthetic liners. The work is well motivated, methodologically detailed, and demonstrates promising agreement between experimental and computational strain estimates. However, the reviewer identifies several important limitations requiring major revision.

Key Strengths

First known attempt to measure liner strain in vivo under realistic loading conditions.

Strong methodological transparency in DIC setup, socket design, and FEA workflow.

Good numerical agreement between DIC and FEA strain patterns, supporting the feasibility of this validation approach.

Major Concerns

•Single participant, limiting generalizability.

•Simplified soft‑tissue modelling (linear elastic instead of hyperelastic).

•Limited DIC field-of-view, restricting strain measurement to a small region.

•Anatomical approximations due to generic bone models and tape‑based scaling.

•Artificial experimental conditions, including lubricant use and glare from the transparent socket.

•Simplified loading conditions with several neglected moments and assumptions.

Required Revisions

•Provide quantitative error metrics between DIC and FEA.

•Justify participant selection and modelling assumptions.

•Expand discussion of optical and mechanical limitations affecting DIC accuracy.

•Clarify why hyperelastic models failed, and better explain strain discrepancies.

•Temper conclusions to reflect the preliminary, single‑subject nature of the study.

Suggested improvement

Lines 19–37

•Comment: The abstract is clear and well structured, but it should acknowledge the single participant design explicitly, as this strongly limits generalizability.

•Suggestion: Add 1–2 sentences describing key limitations upfront to set realistic expectations.

Lines 40–81

•Lines 49–58: You highlight variability in FEA stress predictions due to a lack of validation. This is an excellent justification. However, more explanation of typical error magnitudes or clinical relevance thresholds would strengthen the rationale.

•Line 60: DIC is presented as widely accepted; consider citing more prosthetics-specific DIC applications to position the study within the field.

•Lines 74–81: The two research goals are clearly stated. However, consider emphasising that this is an exploratory single-subject feasibility study, not a validation study.

Lines 85–122

•Lines 85–89: Single participant; demographic justification is absent. Authors should state why this individual was chosen (e.g., typical limb geometry, availability).

•Line 100: Speckle pattern creation using fabric dye and thickener requires justification—does this alter liner compliance or friction properties?

Lines 105–122

•Lines 105–118: The two tasks (vertical loading, half step) are simple and controlled. However, they do not reflect everyday gait dynamics. Authors should justify why a more realistic gait was excluded.

•Lines 118–122: Because only two DIC cameras were available, separate trials were required for anterior and lateral views. This creates potential alignment inconsistencies between datasets and should be acknowledged earlier.

Lines 132–145

•Lines 137–140: More detail is needed on the DIC reconstruction error, stereocalibration accuracy, and noise filtering.

•Lines 140–145: The strain measurement region was extremely limited. This methodological constraint strongly weakens the validity of comparing full field FEA results with partial-field DIC data.

Lines 150–190

•Lines 155–157: Bone anatomy derived from an open-source model and scaled using tape measurements introduces significant geometric uncertainty. Consider quantifying potential anatomical mismatch.

•Lines 166–170: Soft tissues modelled as linear elastic contradict well-established nonlinear, viscoelastic behaviour. A more rigorous rationale is needed.

•Lines 183–190: The loading simplifications (no frontal/transverse plane moments, assumed COP alignment, no inertial effects) reduce ecological validity. Authors should evaluate how these simplifications might bias results.

Lines 199–234

•Lines 200–212: The agreement between DIC and FEA strains is well presented. However, statistical error metrics (e.g., RMSE, correlation, Bland–Altman plots) are missing.

•Lines 211–218: For the half-step task, a substantial discrepancy appears in the lateral minimum and maximum strains. Authors should explain this more clearly rather than briefly noting it.

•Figures: Clear, but inconsistent colour scaling between DIC and FEA maps makes direct visual comparison difficult.

Lines 235–286

•Lines 248–263: The authors compare their strain results to prior DIC studies. Suggest adding discussion on why magnitude differences occur (e.g., liner vs skin measurements).

•Lines 273–278: The explanation of task sensitivity is insightful; however, the discussion should address how the simplified load case compares to actual gait loading.

•Lines 282–285: The authors acknowledge the limitation of linear tissue modelling, but the dismissal of hyperelastic models requires more detail—what specific instabilities occurred?

Lines 288–316

1.Repeatability: No intra-trial variability analysis or repeated measures.

2.Liner nonuniformity: Real prosthetic liners vary locally in thickness and stiffness, unlike the modelled uniform shell.

3.Effect of lubricant and clear socket: The test conditions differ materially from real-world prosthesis use.

4.Region mismatch: The limited DIC area may not correspond perfectly to the FEA region assessed.

Lines 319–324

•Strong concluding summary; however, avoid overgeneralizing given the single participant nature.

•Suggest softening claims about “good agreement,” emphasising that findings are preliminary.

Reviewer #2: Review Comments to the Author:

The manuscript presents a preliminary comparison of prosthetic liner strains measured using digital image correlation (DIC) and predicted using finite element analysis (FEA) in a unilateral transtibial prosthesis user. The study addresses an important and longstanding challenge in prosthetic biomechanics: experimental validation of FEA models used to estimate interface mechanics. The integration of DIC with subject-specific FEA modeling under load-bearing conditions is novel and technically interesting.

Overall, the study demonstrates methodological feasibility and is clearly written. However, several issues related to validation rigor, experimental design, and interpretation must be addressed before the conclusions can be considered adequately supported.

Major Comments:

1. Calibration vs. Validation

The manuscript states that three values of Young’s modulus were evaluated and that 0.3 MPa was selected because it provided the closest match to DIC measurements. However, this approach constitutes calibration rather than independent validation. After tuning material properties to match DIC, the manuscript then concludes that FEA and DIC exhibit “good agreement.”

To strengthen the scientific rigor:

• Clearly distinguish between model calibration and model validation.

• Provide quantitative error metrics (e.g., RMSE, mean absolute error, percent difference, spatial correlation).

• Present strain comparisons for all tested material properties to show how sensitive agreement is to stiffness selection.

• Avoid language implying independent validation unless supported by independent data.

2. Sample Size and Generalizability

The study includes only one participant. While acceptable for a feasibility study, this limits the strength of conclusions. Anatomical variability, tissue properties, and socket fit vary substantially across individuals.

The manuscript should:

• Explicitly frame the study as a single-subject feasibility or proof-of-concept study.

• Avoid generalizable claims regarding prosthetic socket design.

• Clearly acknowledge that findings may be subject-specific.

3. Quantitative Assessment of Agreement

Agreement between DIC and FEA is described largely qualitatively (“good agreement”) and supported by selected peak strain values. However:

• No mesh convergence study is reported for FEA.

• No statistical or spatial comparison metrics are included.

I strongly recommend adding:

• Quantitative comparison metrics.

• A spatial difference map between DIC and FEA strain fields.

• Mesh refinement justification.

4. Modeling Assumptions and Sensitivity

The FEA model includes several simplifying assumptions:

• Linear elastic soft tissues

• Frictionless liner–socket interface

• Simplified loading (sagittal plane only)

• Assumed center-of-pressure alignment

• Non-subject-specific bone geometry

These assumptions may substantially influence strain distribution. While limitations are acknowledged, the manuscript would benefit from:

• A brief sensitivity analysis (e.g., effect of friction coefficient or stiffness variation on strain magnitude).

• Clarification of how loads were applied (to bone nodes, rigid body, distributed load).

• Discussion of how assumptions may bias agreement with DIC.

5. Limited DIC Measurement Region

The measurable strain region is approximately 7 cm in height and 8 cm circumferentially. This represents a limited portion of the liner surface.

The manuscript should:

• Clarify what percentage of total liner surface was analyzed.

• Discuss whether high-strain regions outside this window may have been missed.

• Ensure that claims of agreement are limited to the measured region.

Minor Comments:

• Provide additional DIC processing details (subset size, step size, smoothing, calibration error).

• Ensure identical color scales are used when comparing DIC and FEA strain maps.

• Soften statements such as “validate FEA model results” to reflect the preliminary nature of the study.

• Consider depositing raw DIC data and model inputs in a public repository to align with PLOS data transparency standards.

Reviewer #3: The present study demonstrates the use of DIC to measure prosthesis liner strains of a unilateral transtibial prosthesis user and conducts a preliminary comparison of these strains with those predicted from an FEA model.

The use of DIC has certain challenges as compared to the other experimental techniques, such as:

1. Unstable Speckle Pattern-Sweat or skin stretching alters the speckle pattern, Prosthetic socket friction can erase or distort the pattern, Long walking trials degrade pattern quality. It will be useful mentioning how a Stable Speckle pattern was assured for this study.

2. Secondly Walking involves Dynamic Motion and leads to Motion blurring if cameras are not high speed. Also, Synchronization errors between cameras and gait cycle phases. How are these Issues addressed?

3. Another major drawback of using DIC is that for minor changes in prostheses alignment cause large changes in stress distribution, which makes DIC results difficult to generalize. How is the alignment issue handled in the present study?

4. For a prostheses user, there is a movement of skin moves relative to the underlying bone and muscle known as soft tissue artifact. This phenomenon is known as soft tissue artifact. How present technique addresses this issue and what is the error percentage for the same.

Also, there are some studies done in past that uses DIC for Prostheses Socket Pressure Measurement. Refer to the following study -Evaluation of the influence of cyclic loading on a laser sintered transtibial prosthetic socket using Digital Image Correlation (DIC) by Saey et al. Annu Int Conf IEEE Eng Med Biol Soc, 2019

**********

6. PLOS authors have the option to publish the peer review history of their article (what does this mean?). If published, this will include your full peer review and any attached files.

If you choose “no”, your identity will remain anonymous but your review may still be made public.

**Do you want your identity to be public for this peer review?**  For information about this choice, including consent withdrawal, please see our Privacy Policy.

Reviewer #1: No

Reviewer #2: No

Reviewer #3: **Yes:** Amit Kumar Singh

**********

[NOTE: If reviewer comments were submitted as an attachment file, they will be attached to this email and accessible via the submission site. Please log into your account, locate the manuscript record, and check for the action link "View Attachments". If this link does not appear, there are no attachment files.]

To ensure your figures meet our technical requirements, please review our figure guidelines: https://journals.plos.org/plosone/s/figures

You may also use PLOS’s free figure tool, NAAS, to help you prepare publication quality figures: https://journals.plos.org/plosone/s/figures#loc-tools-for-figure-preparation.

NAAS will assess whether your figures meet our technical requirements by comparing each figure against our figure specifications.

# Author comment: Author response to Decision Letter 1

**DOI:** 10.1371/journal.pone.0353881.r002

28 May 2026

We would like to thank the editor and reviewers for their thoughtful review and constructive comments on our manuscript. We believe that the process of addressing these comments has improved the quality of our manuscript. Our point-by-point responses are indicated by >>>. Changes in the manuscript have been highlighted as requested by the journal.

Journal Requirements:

When submitting your revision, we need you to address these additional requirements.

1. Please ensure that your manuscript meets PLOS ONE's style requirements, including those for file naming. The PLOS ONE style templates can be found at

https://journals.plos.org/plosone/s/file?id=wjVg/PLOSOne_formatting_sample_main_body.pdf and

https://journals.plos.org/plosone/s/file?id=ba62/PLOSOne_formatting_sample_title_authors_affiliations.pdf.

>>> Thank you for sharing these guidelines. We updated our manuscript to adhere to the PLOS ONE style requirements.

2. Please note that PLOS One has specific guidelines on code sharing for submissions in which author-generated code underpins the findings in the manuscript. In these cases, we expect all author-generated code to be made available without restrictions upon publication of the work. Please review our guidelines at https://journals.plos.org/plosone/s/materials-andsoftware-sharing#loc-sharing-code and ensure that your code is shared in a way that follows best practice and facilitates reproducibility and reuse.

>>> Our model input file and data points have been stored in Virginia Tech research repository:

https://doi.org/10.7294/32337195

3. Thank you for stating in your Funding Statement:

“This study was supported by the U.S. Army Medical Research and Development Command under award number: W81XWH-21-1-0220. “

Please provide an amended statement that declares *all* the funding or sources of support (whether external or internal to your organization) received during this study, as detailed online in our guide for authors at http://journals.plos.org/plosone/s/submit-now. Please also include the statement “There was no additional external funding received for this study.” in your updated Funding Statement. Please include your amended Funding Statement within your cover letter. We will change the online submission form on your behalf.

>>> We updated our cover letter.

4. Thank you for stating the following financial disclosure: “This study was supported by the U.S. Army Medical Research and Development Command under award number: W81XWH-21-1-0220. “ Please state what role the funders took in the study. If the funders had no role, please state: "The funders had no role in study design, data collection and analysis, decision to publish, or preparation of the manuscript." If this statement is not correct you must amend it as needed. Please include this amended Role of Funder statement in your cover letter; we will change the online submission form on your behalf.

>>> We updated our cover letter.

5. We note that your Data Availability Statement is currently as follows: [All relevant data are within the manuscript.] Please confirm at this time whether or not your submission contains all raw data required to replicate the results of your study. Authors must share the “minimal data set” for their submission. PLOS defines the minimal data set to consist of the data required to replicate all study findings reported in the article, as well as related metadata and methods (https://journals.plos.org/plosone/s/data-availability#loc-minimal-data-set-definition). For example, authors should submit the following data: - The values behind the means, standard deviations and other measures reported; - The values used to build graphs; - The points extracted from images for analysis. Authors do not need to submit their entire data set if only a portion of the data was used in the reported study. If your submission does not contain these data, please either upload them as Supporting Information files or deposit them to a stable, public repository and provide us with the relevant URLs, DOIs, or accession numbers. For a list of recommended repositories, please see https://journals.plos.org/plosone/s/recommended-repositories. If there are ethical or legal restrictions on sharing a de-identified data set, please explain them in detail (e.g., data contain potentially sensitive information, data are owned by a third-party organization, etc.) and who has imposed them (e.g., an ethics committee). Please also provide contact information for a data access committee, ethics committee, or other institutional body to which data requests may be sent. If data are owned by a third party, please indicate how others may request data access.

>>> Our model input file and data points have been stored in Virginia Tech research repository:

https://doi.org/10.7294/32337195

6. If the reviewer comments include a recommendation to cite specific previously published works, please review and evaluate these publications to determine whether they are relevant and should be cited. There is no requirement to cite these works unless the editor has indicated otherwise. [Note: HTML markup is below. Please do not edit.]

Review Comments to the Author:

Reviewer #1: The manuscript presents a novel and valuable study that compares digital image correlation (DIC) and finite element analysis (FEA) for measuring strain in prosthetic liners. The work is well motivated, methodologically detailed, and demonstrates promising agreement between experimental and computational strain estimates. However, the reviewer identifies several important limitations requiring major revision. Key Strengths First known attempt to measure liner strain in vivo under realistic loading conditions. Strong methodological transparency in DIC setup, socket design, and FEA workflow. Good numerical agreement between DIC and FEA strain patterns, supporting the feasibility of this validation approach.

>>> Thank you for your thorough and constructive review. We appreciate the recognition of the novelty and methodological transparency of our work, and we have addressed each of the concerns and comments raised below.

Major Concerns:

•Single participant, limiting generalizability.

>>>Thank you for raising this concern. We agree that the single-participant design limits generalizability. We have revised the manuscript to explicitly acknowledge this limitation and to clarify that this study is a preliminary feasibility investigation.

•Simplified soft tissue modelling (linear elastic instead of hyperelastic).

>>> We agree that modeling soft tissues as linear elastic is a simplification that does not fully capture their nonlinear behavior. Hyperelastic models were explored; however, they resulted in numerical instabilities under the applied loading conditions and the model did not converge. This issue with hyperelastic models was also reported by Cagle et al. (2018). Due to the focus of this study on feasibility and our limited resources in quantifying subject-specific soft tissue behavior, linear elastic properties were used for modelling soft tissue. We have noted this in the manuscript in the fifth paragraph of the Discussion.

Reference:

“Cagle JC, Reinhall PG, Allyn KJ, McLean J, Hinrichs P, Hafner BJ, et al. A finite element model to assess transtibial prosthetic sockets with elastomeric liners. Medical and Biological Engineering and Computing. 2018;56(7):1227–40.”

Suggested improvement:

Lines 19–37 •Comment: The abstract is clear and well structured, but it should acknowledge the single participant design explicitly, as this strongly limits generalizability. •Suggestion: Add 1–2 sentences describing key limitations upfront to set realistic expectations.

>>> We agree that the single-participant design is an important limitation that should be mentioned upfront. We have revised the abstract to acknowledge this limitation and that the findings are preliminary.

•Lines 49–58: You highlight variability in FEA stress predictions due to a lack of validation. This is an excellent justification. However, more explanation of typical error magnitudes or clinical relevance thresholds would strengthen the rationale.

>>> We have added a sentence to this section noting that FEA stress predictions have been experimentally validated using pressure sensors by other studies, with errors of up to 12 KPa, and that such errors are clinically significant given that repetitive interface stresses as low as 4–23 kPa have been associated with skin damage risk in prosthesis user.(Second paragraph of the Introduction)

•Line 60: DIC is presented as widely accepted; consider citing more prosthetics-specific DIC applications to position the study within the field.

>>> To be clear, DIC is widely accepted in engineering mechanics, but has not been used extensively in prosthesis-related work. In response to the reviewer’s comment, we have made this more clear and included an additional DIC study about prosthetic sockets by Saey et al. (2019). (Third paragraph of the Introduction)

•Lines 74–81: The two research goals are clearly stated. However, consider emphasising that this is an exploratory single subject feasibility study, not a validation study.

>>>We have revised the statement of the goals to explicitly indicate the single-participant and feasibility nature of the study. (Fourth paragraph of the Introduction)

•Lines 85–89: Single participant; demographic justification is absent. Authors should state why this individual was chosen (e.g., typical limb geometry, availability).

>>> The participant was selected based on a) having a below knee amputation at least one year prior; b) having a well-fitting prosthesis that was used daily; and c) schedule availability. We have added this to the paragraph.

•Line 100: Speckle pattern creation using fabric dye and thickener requires justification—does this alter liner compliance or friction properties?

>>> The speckled liner has a rougher surface than normal liner and this will increase the friction coefficient. For this reason, we decided to apply lubricant to remove this effect and have a frictionless liner-socket interface. We have discussed this as a potential limitation in the sixth paragraph of Discussion.

•Lines 105–118: The two tasks (vertical loading, half step) are simple and controlled. However, they do not reflect everyday gait dynamics. Authors should justify why a more realistic gait was excluded.

>>> We agree that selected tasks do not represent full gait dynamics. Full gait was excluded because glare and reflections on the transparent socket were more manageable under-controlled and quasi-static conditions than dynamic walking. Additionally, as a preliminary study, we focused on simple controlled tasks to establish a methodology prior to advancing to more ecologically valid conditions in future work. We have discussed this as a potential limitation in the sixth paragraph of Discussion.

•Lines 118–122: Because only two DIC cameras were available, separate trials were required for anterior and lateral views. This creates potential alignment inconsistencies between datasets and should be acknowledged earlier.

>>> We agree that separate trials for anterior and lateral views could introduce inconsistencies across trials. To minimize the inconsistencies, the participant was provided with clear verbal instructions and visual markers on the forceplate for prosthetic foot positioning. We have added this explanation to the manuscript. We have added this in the third paragraph of Methods.

•Lines 137–140: More detail is needed on the DIC reconstruction error, stereocalibration accuracy, and noise filtering.

>>We have included more information about DIC calibration and filters in the Digital image correlation section.

•Lines 140–145: The strain measurement region was extremely limited. This methodological constraint strongly weakens the validity of comparing full field FEA results with partial-field DIC data.

>>> The measurable DIC region is limited due to proximal sleeve and distal wrap for maintaining the structural integrity of the socket. It is also worth noting that the approximate location of the corresponding region has been shown on FEA results, and all the comparisons were limited to only the corresponding region on the FEA. We acknowledge, however, that this approach limits the ability to draw conclusions about strain distributions across the full liner surface. This issue has been acknowledged as one of the study limitations (Sixth paragraph of the Discussion)

•Lines 155–157: Bone anatomy derived from an open-source model and scaled using tape measurements introduces significant geometric uncertainty. Consider quantifying potential anatomical mismatch.

>>> We agree that our method in bone sizes and positioning could introduce uncertainty. Formal quantification of anatomical mismatch was not possible for us due to lack of imaging data from the participant residual limb bones. We have acknowledged this as a limitation of our study. (Sixth paragraph of the Discussion)

•Lines 166–170: Soft tissues modelled as linear elastic contradict well-established nonlinear, viscoelastic behaviour. A more rigorous rationale is needed.

>>> We agree that linear elastic does not represent the full nonlinear behavior of soft tissues and have acknowledged this as a limitation. Hyperelastic material models were evaluated but could not be used due to solver convergence instability in the FEA software. Cagle et al. (2018) also reported the model convergence instability with hyperelastic models of soft tissues and alternatively adopted a linear elastic model. We have provided more justification in our discussion. (Fifth paragraph of the Discussion)

•Lines 183–190: The loading simplifications (no frontal/transverse plane moments, assumed COP alignment, no inertial effects) reduce ecological validity. Authors should evaluate how these simplifications might bias results.

>>> Neglecting frontal/transverse plane moments could introduce bias of strain values, especially in the lateral side of the residual limb. These moments were neglected following the approach used in prior transtibial prosthetic FEA studies (Cagle et al. (2018) & Mbithi et al. (2022)). Assuming the center of pressure to be aligned with the tibial axis could introduce error in asymmetric strain distribution. Regarding inertial effects, the two loading tasks were intentionally selected quasi-static tasks that were performed slowly to minimize inertial effects, which is consistent with the feasibility scope of this study. We have added more information regarding these limitations to the manuscript. (Sixth paragraph of the Discussion)

•Lines 200–212: The agreement between DIC and FEA strains is well presented. However, statistical error metrics (e.g., RMSE, correlation, Bland–Altman plots) are missing.

>>> Due to differences in mesh discretization between FEA and DIC strain fields, point-wise comparison was not feasible. Gomez et al. (2018) proposed a methodology for such comparisons by comparing strain distribution histograms. In response to this comment, we have adapted their approach and generated probability distribution histograms of principal strains for both FEA and DIC and quantified their overlap using the Bhattacharyya coefficient (BC), a measure of overlap between two probability distributions where a value of 1 indicates complete overlap and 0 indicates no overlap. We additionally compared mean and standard deviation of principal strains between FEA and DIC. Accordingly, we have added a section to our Methods title as “DIC and FEA comparison” and updated our Results to report these comparison measures.

Reference:

Gomez AD, Stone ML, Bayly PV, Prince JL. Quantifying Tensor Field Similarity With Global Distributions and Optimal Transport. Med Image Comput Comput Assist Interv. 2018;11071:428–36.

•Lines 211–218: For the half-step task, a substantial discrepancy appears in the lateral minimum and maximum strains. Authors should explain this more clearly rather than briefly noting it.

>>> We agree that the lateral strain discrepancy during the half-step task needs more explanation. The higher lateral discrepancy between FEA and DIC during the half-step task could b

Attachment Submitted filename: Response to reviewers.docx

# Peer review: Decision Letter 1

**DOI:** 10.1371/journal.pone.0353881.r003

**Authors:** Alessandra Aldieri, Alessandra Aldieri

30 Jun 2026

A preliminary comparison of prosthetic socket liner strain determined using digital image correlation and finite element analysis

PONE-D-26-07309R1

Dear Dr. Philen,

We’re pleased to inform you that your manuscript has been judged scientifically suitable for publication and will be formally accepted for publication once it meets all outstanding technical requirements.

Within one week, you’ll receive an e-mail detailing the required amendments. When these have been addressed, you’ll receive a formal acceptance letter and your manuscript will be scheduled for publication.

An invoice will be generated when your article is formally accepted. Please note, if your institution has a publishing partnership with PLOS and your article meets the relevant criteria, all or part of your publication costs will be covered. Please make sure your user information is up-to-date by logging into Editorial Manager at Editorial Manager® and clicking the ‘Update My Information' link at the top of the page. For questions related to billing, please contact billing support.

If your institution or institutions have a press office, please notify them about your upcoming paper to help maximize its impact. If they’ll be preparing press materials, please inform our press team as soon as possible -- no later than 48 hours after receiving the formal acceptance. Your manuscript will remain under strict press embargo until 2 pm Eastern Time on the date of publication. For more information, please contact onepress@plos.org.

Kind regards,

Alessandra Aldieri

Academic Editor

PLOS One

Additional Editor Comments (optional):

Reviewers' comments:

Reviewer's Responses to Questions

**Comments to the Author**

1. If the authors have adequately addressed your comments raised in a previous round of review and you feel that this manuscript is now acceptable for publication, you may indicate that here to bypass the “Comments to the Author” section, enter your conflict of interest statement in the “Confidential to Editor” section, and submit your "Accept" recommendation.

Reviewer #1: All comments have been addressed

Reviewer #2: All comments have been addressed

**********

2. Is the manuscript technically sound, and do the data support the conclusions?

The manuscript must describe a technically sound piece of scientific research with data that supports the conclusions. Experiments must have been conducted rigorously, with appropriate controls, replication, and sample sizes. The conclusions must be drawn appropriately based on the data presented.

Reviewer #1: Yes

Reviewer #2: Yes

**********

3. Has the statistical analysis been performed appropriately and rigorously?

Reviewer #1: Yes

Reviewer #2: Yes

**********

4. Have the authors made all data underlying the findings in their manuscript fully available?

The PLOS Data policy requires authors to make all data underlying the findings described in their manuscript fully available without restriction, with rare exception (please refer to the Data Availability Statement in the manuscript PDF file). The data should be provided as part of the manuscript or its supporting information, or deposited to a public repository. For example, in addition to summary statistics, the data points behind means, medians and variance measures should be available. If there are restrictions on publicly sharing data—e.g. participant privacy or use of data from a third party—those must be specified.

Reviewer #1: Yes

Reviewer #2: Yes

**********

5. Is the manuscript presented in an intelligible fashion and written in standard English?

PLOS ONE does not copyedit accepted manuscripts, so the language in submitted articles must be clear, correct, and unambiguous. Any typographical or grammatical errors should be corrected at revision, so please note any specific errors here.

Reviewer #1: Yes

Reviewer #2: Yes

**********

6. Review Comments to the Author

Please use the space provided to explain your answers to the questions above. You may also include additional comments for the author, including concerns about dual publication, research ethics, or publication ethics. (Please upload your review as an attachment if it exceeds 20,000 characters)

Reviewer #1: Thank you for addressing the reviewers' comments. The rebuttals to the reviewer comments are itemized individually. The inclusion of the document with track changes enabled the reviewers to verify the actions taken by the authors in response to their comments.

Reviewer #2: The authors have responded thoroughly and constructively to the comments raised during the initial review. The revised manuscript has been substantially improved, particularly in the following aspects:

* The study is now appropriately framed as a preliminary, single-participant feasibility investigation rather than an independent validation study.

* Quantitative comparison metrics between DIC and FEA have been added, including distribution-based analyses and supporting sensitivity studies.

* Data and model inputs have been made publicly available in accordance with the journal's data-sharing requirements.

* The limitations of the methodology and the assumptions underlying the finite element model are now clearly acknowledged and discussed.

* The conclusions have been appropriately softened to reflect the preliminary and subject-specific nature of the findings.

While some limitations remain, they are inherent to the feasibility nature of the study and have been transparently addressed by the authors. I do not believe that further revision would substantially improve the scientific contribution of the manuscript.

In my opinion, the manuscript is technically sound, the conclusions are appropriately supported by the data presented, and the work provides a novel and valuable methodological contribution to the field of prosthetic biomechanics.

**********

7. PLOS authors have the option to publish the peer review history of their article (what does this mean?). If published, this will include your full peer review and any attached files.

If you choose “no”, your identity will remain anonymous but your review may still be made public.

**Do you want your identity to be public for this peer review?**  For information about this choice, including consent withdrawal, please see our Privacy Policy.

Reviewer #1: No

Reviewer #2: **Yes:** Norwahida Yusoff

**********

# Editor report: Acceptance letter

**DOI:** 10.1371/journal.pone.0353881.r004

**Authors:** Alessandra Aldieri, Alessandra Aldieri

PONE-D-26-07309R1

PLOS One

Dear Dr. Philen,

I'm pleased to inform you that your manuscript has been deemed suitable for publication in PLOS One. Congratulations! Your manuscript is now being handed over to our production team.

At this stage, our production department will prepare your paper for publication. This includes ensuring the following:

* All references, tables, and figures are properly cited

* All relevant supporting information is included in the manuscript submission,

* There are no issues that prevent the paper from being properly typeset

You will receive further instructions from the production team, including instructions on how to review your proof when it is ready. Please keep in mind that we are working through a large volume of accepted articles, so please give us a few days to review your paper and let you know the next and final steps.

Lastly, if your institution or institutions have a press office, please let them know about your upcoming paper now to help maximize its impact. If they'll be preparing press materials, please inform our press team within the next 48 hours. Your manuscript will remain under strict press embargo until 2 pm Eastern Time on the date of publication. For more information, please contact onepress@plos.org.

You will receive an invoice from PLOS for your publication fee after your manuscript has reached the completed accept phase. If you receive an email requesting payment before acceptance or for any other service, this may be a phishing scheme. Learn how to identify phishing emails and protect your accounts at https://explore.plos.org/phishing.

If we can help with anything else, please email us at customercare@plos.org.

Thank you for submitting your work to PLOS ONE and supporting open access.

Kind regards,

PLOS ONE Editorial Office Staff

on behalf of

Dr. Alessandra Aldieri

Academic Editor

PLOS One
